# Supplementary material for: Improving equity in access to early diagnosis of cancer in different healthcare systems of Latin America: protocol for the EquityCancer-LA implementation-effectiveness hybrid study
Source: BMJ Open. 2022 Dec 13;12(12):e067439. doi: 10.1136/bmjopen-2022-067439 (PMC9748968; doi:10.1136/bmjopen-2022-067439)
Supplement: Supplementary data [file bmjopen-2022-067439supp001.pdf]

Supplemental Figure 1. Theoretical framework for the analysis of pathways to diagnosis of cancer and influencing factors in LA

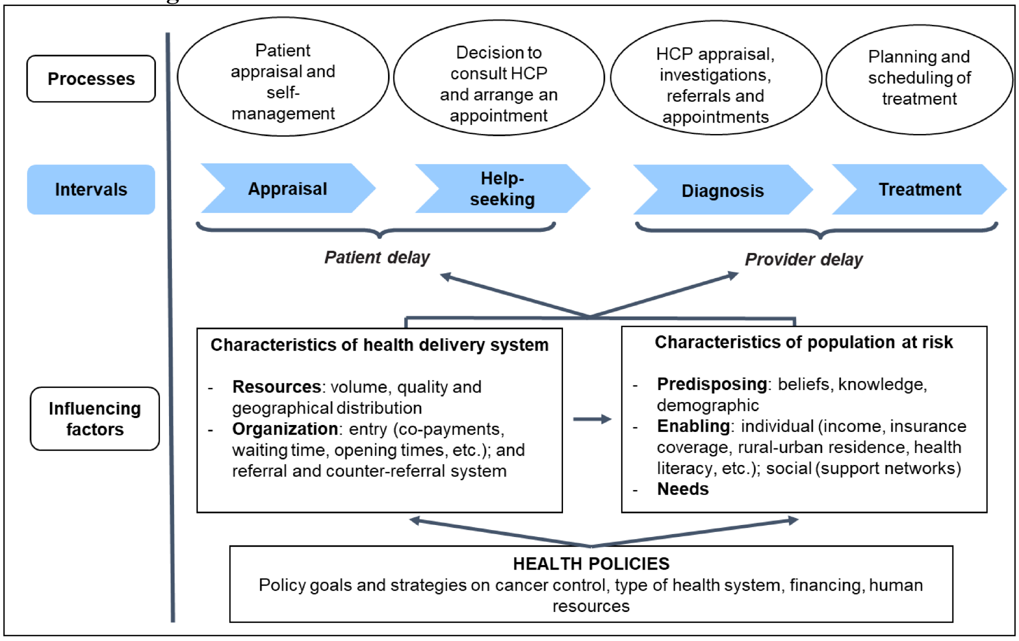

HCP: Healthcare provider, Source: Modified from Walter et al. (2012) [75] based on Aday&Andersen[79]
