# Supplementary material for: Improving equity in access to early diagnosis of cancer in different healthcare systems of Latin America: protocol for the EquityCancer-LA implementation-effectiveness hybrid study
Source: BMJ Open. 2022 Dec 13;12(12):e067439. doi: 10.1136/bmjopen-2022-067439 (PMC9748968; doi:10.1136/bmjopen-2022-067439)
Supplement: Author's manuscript [file bmjopen-2022-067439.draft_revisions.pdf]

BMJ Open

BMJ Open is committed to open peer review. As part of this commitment we make the peer review history of every article we publish publicly available.

When an article is published we post the peer reviewers' comments and the authors' responses online. We also post the versions of the paper that were used during peer review. These are the versions that the peer review comments apply to.

The versions of the paper that follow are the versions that were submitted during the peer review process. They are not the versions of record or the final published versions. They should not be cited or distributed as the published version of this manuscript.

BMJ Open is an open access journal and the full, final, typeset and author-corrected version of record of the manuscript is available on our site with no access controls, subscription charges or pay-per-view fees (http://bmjopen.bmj.com).

If you have any questions on BMJ Open's open peer review process please email info.bmjopen@bmj.com

BMJ Open

Improving equity in access to early diagnosis of cancer through implementation research in different healthcare systems of Latin America: the EquityCancer-LA study protocol

Journal:	*BMJ Open*
Manuscript ID	bmjopen-2022-067439
Article Type:	Protocol
Date Submitted by the Author:	16-Aug-2022

Complete List of Authors:	Vazquez, Maria-Luisa; Research Services and Prospectives on Health Policy of Health and Social Consortium of Catalonia<br>Vargas, Ingrid; Research Services and Prospectives on Health Policy of Health and Social Consortium of Catalonia<br>Rubio-Valera, Maria; Parc Sanitari Sant Joan de Déu, Centro de Investigación Biomédica en Red de Epidemiología y Salud Pública (CIBERESP)<br>Aznar-Lou, Ignacio; Institut de Recerca Sant Joan de Deu, Centro de Investigación Biomédica en Red de Epidemiología y Salud Pública (CIBERESP).Research and Development Unit<br>Eguiguren, Pamela; Universidad de Chile, Escuela de Salud Pública Dr. Salvador Allende. Facultad de Medicina<br>Mogollón-Pérez, Amparo S; Universidad del Rosario, Escuela de Medicina y Ciencias de la Salud<br>Torres-Castillo, Ana Lucía; Pontificia Universidad Católica del Ecuador, Public Health Institute<br>Peralta, Andrés; Pontificia Universidad Católica del Ecuador, Public Health Institute<br>Dias, Sónia; Nova University of Lisbon, Comprehensive Health Research Center (CHRC). NOVA National School of Public Health, Public Health Research Centre<br>Jervelund, Signe; University of Copenhagen, Health Services Research

Keywords:	Change management < HEALTH SERVICES ADMINISTRATION & MANAGEMENT, Organisation of health services < HEALTH SERVICES ADMINISTRATION & MANAGEMENT, Quality in health care < HEALTH SERVICES ADMINISTRATION & MANAGEMENT, MEDICAL EDUCATION & TRAINING, Adult oncology < ONCOLOGY, Health policy < HEALTH SERVICES ADMINISTRATION & MANAGEMENT

SCHOLARONE™
Manuscripts

Improving equity in access to early diagnosis of cancer through implementation research in different healthcare systems of Latin America: the EquityCancer-LA study protocol

Corresponding author:
Ingrid Vargas
Servei d'Estudis i Prospectives en Polítiques de Salut
Consorci de Salut i Social de Catalunya
Avinguda Tibabo 21
08022 Barcelona
Tel. 00-34-932531820
ivargas@consorci.org

María-Luisa Vázquez
Health Policy and Health Services Research Group, Health Policy Research Unit, Consortium for Health Care and Social Services of Catalonia
Avinguda Tibidabo, 21
08022 Barcelona, Spain
mlvazquez@consorci.org

Ingrid Vargas
Health Policy and Health Services Research Group, Health Policy Research Unit, Consortium for Health Care and Social Services of Catalonia
Avinguda Tibidabo, 21
08022 Barcelona, Spain
ivargas@consorci.org

Maria Rubio-Valera
Centro de Investigación Biomédica en Red de Epidemiología y Salud Pública (CIBERESP)
Parc Sanitari Sant Joan de Déu,
Doctor Antoni Pujadas 42,
Sant Boi de Llobregat, Barcelona, España
maria.rubio@sjd.es

Ignacio Aznar-Lou
Centro de Investigación Biomédica en Red de Epidemiología y Salud Pública (CIBERESP)
Research and Development Unit, Institut de Recerca Sant Joan de Déu
Santa Rosa 39-57
Esplugues de Llobregat, Barcelona, España
ignacio.aznar@sjd.es

For peer review only - http://bmjopen.bmj.com/site/about/guidelines.xhtml

Pamela Eguiguren
Escuela de Salud Pública Dr. Salvador Allende Gossens, Facultad de Medicina, Universidad de Chile
Avenida Independencia, 939
Santiago de Chile, Chile
peguiguren@u.uchile.cl

Amparo-Susana Mogollón-Pérez
Escuela de Medicina y de Ciencias de la Salud, Universidad del Rosario
Carrera 24 No. 63 C-69. Quinta Mutis
Bogotá, Colombia
amparo.mogollon@urosario.edu.co

Ana-Lucía Torres
Public Health Institute, Pontifical Cathloic University of Ecuador
Av. 12 de Octubre, 1076, Vicente Ramón Roca
Quito, Ecuador
atorres331@puce.edu.ec

Andrés Peralta
Public Health Institute, Pontifical Cathloic University of Ecuador
Av. 12 de Octubre, 1076, Vicente Ramón Roca
Quito, Ecuador
aaperalta@puce.edu.ec

Sónia Dias
NOVA National School of Public Health, Public Health Research Centre, NOVA University of Lisbon & Comprehensive Health Research Center (CHRC)
Av. Padre Cruz, 1600-560
Lisboa, Portugal
sonia.dias@ensp.unl.pt

Signe Smith Jervelund
Department of Public Health, Faculty of Health and Medical Sciences, University of Copenhagen
Øster Farimagsgade 5
Copenhagen, Denmark
ssj@sund.ku.dk

for EquityCancer-LA

For peer review only

KEY WORDS

Cancer early diagnosis; integrated care interventions; equity in access; implementation research; participatory action research; Latin America

Word count: 3,871

For peer review only

For peer review only - http://bmjopen.bmj.com/site/about/guidelines.xhtml

Improving equity in access to early diagnosis of cancer through implementation research in different healthcare systems of Latin America: the EquityCancer-LA study protocol

ABSTRACT

Introduction

Healthcare fragmentation is a main cause for delay in cancer diagnosis and treatment, contributing to high and steadily increasing mortality rates in Latin America (LA), particularly among disadvantaged populations. This research focuses on integrated care interventions, which have proven effective in improving early diagnosis of cancer and are also promoted by national cancer plans in LA, but limitedly implemented or evaluated in the region. The objective of the study is to evaluate the contextual effectiveness of scaling-up a multi-component integrated care intervention to improve early diagnosis of frequent cancers in healthcare networks of Chile, Colombia and Ecuador.

Methods and analysis

This participatory, interdisciplinary and mix-methods implementation research is two-pronged: a) a quasi-experimental design (controlled before and after) with an intervention and a control healthcare network in each LA country, using an implementation-effectiveness hybrid approach to assess the intervention implementation process, contextual effectiveness, and costs; b) a case study design for the in-depth analysis of access to cancer diagnosis of most frequent cancers. Focussing on the most vulnerable socioeconomic population, it develops in four phases: 1) analysis of delays, related factors and contextual barriers to early diagnosis (base-line); 2) adaptation and implementation of the multi-component integrated care intervention to improve early diagnosis (PC training, fast-track referral pathway and patient information). A participatory action research approach will be used to tailor the

For peer review only - http://bmjopen.bmj.com/site/about/guidelines.xhtml

intervention to each context, led by a local steering committee (healthcare network professionals, managers, insurers (Colombia), policy-makers, patients and researchers); 3) intra-country evaluation of intervention; 4) cross-country analysis.

Ethics and dissemination

This study complies with international and national legal stipulations on ethics. Conditions of the study procedure were approved by each country's ethical committee. A variety of dissemination activities are implemented addressing the main stakeholders. Registration No.: 11878848 in Universidad del Rosario, Colombia; and 030-UIO-2021 in PUCE, Ecuador.

STRENGTHS AND LIMITATIONS

- EquityCancer-LA represents the first initiative in the Latin American region, and one of the first at international level, to evaluate the effectiveness and the implementation process of a multi-component integrated care intervention to improve early diagnosis of cancer under real-life conditions.
- This research will provide evidence on the adoption of a participatory approach to adapt and implement (scale-up) an effective intervention for early diagnosis of cancer.
- EquityCancer-LA will provide evidence and recommendations to national programmes and policies focusing on early diagnosis and a validated strategy for the large-scale implementation of the intervention in LA and LMICs.
- This research will generate evidence on key barriers and facilitators that determine delays in diagnosis of cancer in healthcare networks in different health systems of three LA countries.
- A potential limitation might be the political changes during the intervention implementation process, leading to changes in managerial positions of participant healthcare networks and to a lack of institutional support for the interventions.

For peer review only

For peer review only - http://bmjopen.bmj.com/site/about/guidelines.xhtml

Collaboration agreements will be signed with the healthcare networks at the beginning of the project, and a fluid communication will be pursued to guarantee a full commitment.

INTRODUCTION

Delays in diagnosis and treatment contribute to the high and steadily increasing cancer mortality rates in Latin America (LA)[1–4]. WHO recently established early cancer diagnosis programmes as a priority for low and middle income countries (LMICs) [5–7] and defined it as the early identification of cancer in patients who have symptoms of the disease [5]. The objective of early cancer diagnosis- which is the focus of EquityCancer-LA- is to identify the disease in people who have symptoms and signs consistent with cancer at the earliest possible opportunity and to link it with treatment without delay [5]. Interventions to ensure adequate and timely access and strong care coordination across the required health services (primary care, diagnostic and specialist services) are key for cancer control. Early diagnosis and referral have been affected by the COVID-19 outbreak responses (confinement, resources diversion and interruption of screening programs, etc.), making interventions in this area more relevant than ever[8–10].

The patient pathway from the discovery of symptoms to a confirmed diagnosis of cancer is often very complicated, encompassing different healthcare levels, services and professionals and contributing to delays in treatment. While delays may differ according to patient and healthcare system characteristics, for its analysis (and to define interventions to address them) two types of intervals are generally distinguished[11]: the *patient interval*, or time taken from the discovery of symptoms to seeking a medical professional, and the *provider interval*, from the request of the first medical consultation to the confirmation of cancer diagnosis.

Delays in cancer diagnosis are accentuated in fragmented healthcare systems such as those in LA, characterized by limited access to health services and poor coordination of care among a

For peer review only - http://bmjopen.bmj.com/site/about/guidelines.xhtml

wide range of providers. Although further research is required, available studies in LA suggest that most delays occur within the provider interval [4,13,14] and are related to financial, geographic and organizational barriers of access, including poor diagnostic suspicion in primary care (PC) and limited cross-level care coordination [3,15,16]. Population groups most affected are those of low socioeconomic status living in large urban slums, rural inhabitants, indigenous populations [3], or those on the subsidized scheme in Colombia (no formal employment)[17], resulting in large inequities in early diagnosis [2,18].

Integrated care as a strategy to improve early cancer diagnosis

The implementation of integrated care interventions focusing on strengthening PC and improving care coordination across care levels are considered essential [19–21]. This is particularly so in PC-based health systems –as in most Latin American countries [22] – in which PC is the entry point to health care and where, around 85% of cancers are diagnosed after symptomatic presentation to PC [19], including cancers for which screening programmes exists [23]. Therefore, to improve the early diagnosis of cancer, PC doctors should be able to assess the probability of cancer, and to refer rapidly suspected cancer patients to specialized care for cancer investigations and treatment [19,23]. To this end, comprehensive multi-component interventions, combining PC training with improved diagnostic and treatment pathways and patient information to navigate the health services, are increasingly recommended [24], also for low and middle income countries (LMICs) [5–7], but introduced mainly in high income countries (HICs) [23,25–29]. In LA, current national cancer plans and strategies [30–35] promote integrated cancer care strategies to address fragmentation and geographic inequity in cancer diagnosis and care. However, the implementation of these integrated care strategies is very limited, their evaluation scarce [36–38] and focused on specific mechanisms [39–42].

The effectiveness of integrated care as a strategy to improve early cancer diagnosis

For peer review only - http://bmjopen.bmj.com/site/about/guidelines.xhtml

Available evidence, mostly from HICs, shows that the implementation of continuing education on early diagnosis of PC teams, fast-track referral pathways and guidelines and patient information to navigate the health services are effective [23,26,27,43–45] and efficient [23,46–48] in improving early diagnosis of cancer. Integrated care strategies can improve the appropriateness of referrals and tests and increase diagnostic accuracy for PC doctors [23,43,49], which can in turn reduce the provider delay [19,27,50]. Moreover, the use of information and communication technology (ICT) in PC training (e.g. videoconferencing) and virtual clinical consultation show multiple benefits (e.g. increased access to secondary/tertiary care (SC/TC), care coordination across levels, etc.) especially in big cities with traffic problems and rural areas [51–55].

Evidence also shows that interventions are only successful if adapted and implemented taking into account local problems (delays, pathways, barriers) [15], priorities and organizational factors and are agreed upon by the professionals involved across care levels [56–62]. In this respect, participatory and multidisciplinary approaches, including participatory action research (PAR) has developed a growing reputation as an effective means for tailoring interventions and achieving organizational change [63], although its application to improve early diagnosis of cancer has been rare [64]. PAR main characteristics are: non-linear and reflexive process, collaboration between local actors and researchers, involvement of the local stakeholders throughout the process, country research teams as facilitators (capacity building, systematization, monitoring and feedback), and based on three basic principles, egalitarian participation, shared decision-making and co-production of knowledge[65]. Involving main actors lends greater contextual relevance and validity to interventions, creates more interest and increases sustainability [65,66]. Moreover, participatory training methods improve not only knowledge and skills but also favour coordination between professionals and stakeholders throughout the healthcare network[67].

For peer review only

The need for implementation research in Latin America

Understanding the extent to which an effective intervention for early diagnosis of cancer is so also in its specific health systems and social contexts, is challenging but needed and calls for implementation research. This approach helps to determine the factors of success or failure in the intervention implementation related to the context and the process, including the keys to its sustainability, affordability and large scale use [68,69]. However, limited implementation research has been conducted in LMICs, including LA [67,70] - and even less to evaluate interventions for early diagnosis of cancer.

Aim of the EquityCancer-LA project

The aim of EquityCancer-LA is to evaluate the contextual effectiveness of scaling-up a multi-component integrated care intervention to improve early diagnosis of cancer, taking a participatory approach, in healthcare networks of different healthcare systems in Latin America. This project builds upon frameworks, results, and best practices in implementing integrated care interventions using a PAR approach, generated by Equity-LA[71] and Equity-LA II[67] research projects. This paper outlines the study protocol of EquityCancer-LA.

METHODS AND ANALYSIS

Study design

EquityCancer-LA adopts a double design: a) a quasi-experimental design (a control before and after design) with an intervention and a control health services network in each LA country, using a participatory and implementation-effectiveness hybrid approach[68] to assess the intervention implementation process, contextual effectiveness , and costs and b) a case study design [72], using mix-methods [73], for the in-depth analysis of key barriers and facilitators to early cancer diagnosis of most frequent cancers and its implications for equity of access.

For peer review only - http://bmjopen.bmj.com/site/about/guidelines.xhtml

Analytical frameworks adapted for the evaluation of the intervention contextual effectiveness and analysis of access to early diagnosis of cancer

This study is underpinned by two conceptual frameworks:

I) Analytical framework for the evaluation of an intervention contextual effectiveness and implementation process (Figure 1), based on the Equity-LA II developed framework [67]. It includes a) the evaluation of intervention effectiveness and cost-effectiveness based on intermediate and final outcomes related to cancer diagnosis; and b) the analysis of the interventions process and how it affects its effectiveness, embracing the three dimensions of Pettigrew and Whipp [69,74]: the context, the process of design and implementation, and the content of the intervention. The evaluation of the costs of the implementation, as part of the analysis of the intervention process, will be oriented by the SIC framework[75].

II) Framework for the analysis of access to early diagnosis of cancer, built upon the model for the analysis of pathways to cancer diagnosis and treatment by Walter et al. (2012) [76], and incorporating factors influencing healthcare access, as proposed by Aday and Andersen [77–79] (online supplemental Figure 1). According to this framework, delays throughout the patient trajectory to confirmation of cancer diagnosis and initiation of treatment can be influenced by characteristics of the population at risk: need, predisposing (sociodemographic, believes, attitudes, knowledge) and enabling (income, health insurance, residence, etc.) factors, as well as health services' factors: resources availability and organization of health services (fee for service, waiting times, schedules, referral system, etc.) [12]. Health policies can in turn affect access barriers related to the health services or changing (mutable) characteristics of the population at risk [12].

Study context

The field work of EquityCancer-LA is being carried out in three LA countries, Chile, Colombia and Ecuador, and within them, in two public health services networks providing

For peer review only - http://bmjopen.bmj.com/site/about/guidelines.xhtml

care to middle-low and low income areas. Although the study countries have different health system models, all are segmented by population groups according to socioeconomic or employment status [80,81], with a public subsystem and a private one. The public sector, which is the focus of this study, is financed by social security contributions and/or taxes. It encompasses at least one subsystem dependent on the ministry of health, which is decentralized to different levels of government (departments/provinces and/or municipalities) and is generally aimed at the lower income population and/or those without social security. In each country, two comparable health services networks (one intervention (IN) and one control (CN)) have been selected according to the following criteria: a) provision of a continuum of services including at least PC and SC/TC, responsible for diagnosis and treatment of cancer; b) provision of services to a defined population; c) provision of care mainly to urban low and middle low socioeconomic areas; d) willingness to participate and implement the intervention; and e) leadership with competence for implementing the intervention. Selected networks are: in Chile, the northern network (IN) and southern network (CN) of Santiago; in Colombia, two networks of Cundinamarca, encompassing 14 health regions; in Ecuador, 17D06 (IN) and 17D03 (CN) districts in the southern and northern areas of Quito.

Research phases and methods

EquityCancer-LA is structured in four research phases which extends over 60 months (May 2021- April 2026) and adopts an interdisciplinary, participatory and mix-methods approach (Figure 2). The phases are: (1) an analysis of key barriers and facilitators for early diagnosis (baseline study), using both qualitative and quantitative methods, which is currently underway; (2) participatory adaptation and implementation of the intervention; (3) monitoring and evaluation of the intervention; and a (4) cross-country comparative analysis. At the beginning of the project, a local steering committee (LSC) of relevant stakeholders - health professionals from different care levels, managers, healthcare insurers (in Colombia), policy-

For peer review only - http://bmjopen.bmj.com/site/about/guidelines.xhtml

makers, patients and the research team - was established in each country. It will be involved in all research phases and will lead the adaptation of the intervention, implementation process, and dissemination of results.

I. ***Analysis of key barriers and facilitators of early cancer diagnosis*** of the most frequent cancers (base-line) ***and implications for equity of access***. During the first six months of this phase, the theoretical framework and research plan were finalized, and on the basis of available data, the LSCs selected frequent cancers to be addressed: breast, cervical, colorectal, stomach, lung and prostate cancers. The next step is to analyse the access to diagnosis of cancer in the study networks. This analysis employs a mixed methods approach, combining qualitative and quantitative research methods of social sciences for data collection in an iterative and complementary way.

a) A qualitative study to analyse barriers and facilitators of access and patient's pathways to early diagnosis in each particular context from the stakeholders' perspective. A criterion sample was designed to include all discursive variants on the phenomenon: health professionals, administrative personnel, managers, policy makers, and cancer patients, with particular focus on vulnerable groups (low socioeconomic status, gender, ethnicity, distance to SC/TC services and rurality). Data collection takes place in two phases, before and after the patients' questionnaire survey, using focus groups (FG), semi-structured individual interviews and policy documents' analysis. A thematic analysis has been conducted with support of a specialized software (Atlas-ti, NVivo) to identify main contextual barriers and facilitators of cancer diagnosis to inform the adaptation of the intervention in each area. In the second phase, critical cases identified by the patient survey will be invited to become case studies for an in-depth analysis to better capture patient journey complexity, reasons for delays in the diagnostic process as well as health consequences.

For peer review only - http://bmjopen.bmj.com/site/about/guidelines.xhtml

b) A quantitative study. A cross-sectional study by means of a questionnaire survey of patients diagnosed of cancer will be conducted in each country to establish differences in use of health services and pathways in cancer diagnosis, time intervals, barriers to early diagnosis among different population groups, costs, and where possible, complemented with PC and SC/TC medical records audits. It will provide also the base-line for the evaluation of the intervention, to compare both networks before and after, and also the intervention and control networks. The study population will comprise patients aged 18 years or more with a confirmed cancer diagnosed and staged in the 3–6 months prior to the survey in the study healthcare networks. Patients with benign tumour, uncertain, with consecutive cancers in same organ (recurrence) or synchronous primary cancers will be excluded. The sample size of the study population has been estimated taking into account the controlled before and after design of the study and in order to ensure the detection of a 15% variation in time intervals, health services use and barriers to the early diagnosis of cancer in a bilateral contrast (before and after, intervention and control). A sample size of 174 patients with a diagnosis of cancer per healthcare network and year has been estimated, on the basis of a power of 80% ($\beta = 0.20$) and a confidence level of 95% ($\alpha = 0.05$). It will be selected from the patient data bases. The results will contribute to adapt the intervention to the context, to analyse costs, barriers and inequalities in access to early diagnosis of cancer. The survey started in August 2022.

II. ***Participatory adaptation and implementation of the intervention*** to improve early diagnosis of frequent cancers in the countries of study, under real-life conditions.

EquityCancer-LA will adapt, scale-up and assess the feasibility of an effective, innovative and comprehensive integrated care intervention to reduce the provider delay(interval) in the diagnosis of the most frequent cancers. The components of the intervention are: a) *PC doctor's training* to improve PC early diagnosis capacity and care

For peer review only - http://bmjopen.bmj.com/site/about/guidelines.xhtml

For peer review only

coordination across care levels. According to the context, it will be on-line (videoconferencing) or face-to-face, and reflexive and participatory methods will be used; b) a fast-track referral pathway to expedite referral for diagnostic evaluation of patients jointly designed by PC and SC doctors, supported by the description of alarm symptoms that may raise cancer suspicion, standardised pathways or circuits for the diagnostic process and initial treatment, and referral guidelines agreed upon by PC and SC levels' doctors; and c) a patient information strategy to assist the patients to navigate the system. The three components interact reinforcing each other effectiveness (Figure 3). The intervention has been conceived based on available evidence, WHO recommendations for LMICs [5] and national cancer plans [30–32].

A participatory action research approach (PAR) will be used to tailor the multi-component integrated care intervention to each context and implement it based on the preliminary results of the base-line study and the assessment of the implementation conditions, involving main stakeholders and building on previous Equity-LA II [67,82] experience and methods. A carefully phased approach will be used (Figure 4). The participatory process starts in each LA country with the creation of the LSC. First, the evidence produced on the barriers and facilitators of early diagnosis of cancer and previous experiences related to the intervention in the healthcare networks will discussed with the LSC and the inter-level group of professionals (GP), which will be created with those interested in taking action. The conditions for the intervention implementation (acceptability, appropriateness, feasibility, costs) and contextual barriers and facilitators will be evaluated with relevant stakeholders to introduce any necessary changes prior to its implementation. In Chile, an evaluation will be made of the conditions for the extension to the whole network of the virtual inter-level consultations that were participatory designed and implemented in the framework of the Equity-LA II project.

For peer review only - http://bmjopen.bmj.com/site/about/guidelines.xhtml

In the second phase, the different components of the intervention will be adapted by the GP and LSC (contents, method, implementation plan and monitoring indicators) and some of them will be pilot-tested, if relevant. Inter-levels working groups might be established for the adaptation of specific components, made up of participants from the different care levels and departments involved in the cancer care process. In this phase, the definitive plan for the intervention evaluation will be elaborated (including feasible and valid measures of intermediate and final outcomes, etc.).

III. ***Monitoring and evaluation of the intervention*** will address both, the implementation process, as well as, the contextual effectiveness, including the economic evaluation (cost-effectiveness and implementation costs), in each country to determine its sustainability and applicability to other contexts. *Monitoring the intervention* during the implementation process will facilitate its adaptation, the identification and correction of any contextual factors that may influence its implementation and the costs analysis. Indicators will be elaborated to monitor the implementation process (uptake, costs, etc.) and to identify trends in effectiveness regarding intermediate and, if possible, final outcomes.

The *evaluation of the implementation process* will identify the factors related to the context, process and intervention content that have influenced the implementation and its effectiveness, as well as its sustainability and replicability to other contexts. It will be based mainly on a qualitative study, which will be essential to discriminate what component of the intervention was responsible for what results. Individual interviews and focus groups will be conducted with different stakeholders involved in the process. These are: LSC, inter-level group of professionals, managers, policy-makers, and patients. This study will be complemented with quantitative analyses of specific aspects: uptake, acceptability, appropriateness regarding specific problems in cancer early diagnosis (process indicators; doctor survey).

For peer review only - http://bmjopen.bmj.com/site/about/guidelines.xhtml

The *evaluation of the effectiveness* will encompass two intra-country comparison (before and after the intervention in each network and between the intervention and control networks). To identify any changes after the intervention, data will be collected by: a) a pre- post- survey to participant doctors, b) the patient survey and medical records audit (when possible), using the methods and instruments of the base-line, and c) monitoring indicators. Impact on PC doctor's knowledge will be measured applying an anonymous on-line questionnaire survey before the training starts (base-line), at the end and eight months after to measure any improvement in knowledge. Final outcome measures will be defined after the adaptation and testing of the intervention and may include intermediate healthcare outcomes (pathways in cancer diagnosis, time intervals, barriers of access) and final outcomes (provider delays and stage of cancer at diagnosis).

Two kinds of *economic analysis* will be carried out: a) the implementation cost to estimate total costs of the implementation strategy; and b) a cost-effectiveness analysis of the intervention to calculate incremental costs per reduced diagnostic delay.

The evaluation will be completed combining the results of qualitative and quantitative studies, to identify the contextual effectiveness of the intervention on early diagnosis of cancer and influencing factors in each country.

IV. ***Cross-country comparative analysis***. Results will be compared across countries to identify contextual factors and actors that influence, on the one hand, access and equity of access to early diagnosis of cancer in different healthcare systems, and on the other, the process and effectiveness results of the intervention; and, to inform the strategy to embed the intervention into local, regional and national health policy and practice.

To ensure these components develop according to plan and to an excellent level of quality and maximum societal impact, coordination and management activities, as well as activities for dissemination and exploitation of results and capacity building will run in parallel and will

For peer review only - http://bmjopen.bmj.com/site/about/guidelines.xhtml
For peer review only

count on the involvement of the LSCs and the support of national and international scientific and international ethics advisory committee.

Patient and public involvement

Patients are part, together with other key stakeholders, of the LSC and WG involved in the adaptation and implementation of the intervention, and specifically, of the component related to the patient information strategy to navigate the health services. They will also be involved in the dissemination of results.

ETHICS AND DISSEMINATION

Ethical issues

The development and execution of the project fully complies with all current international conventions and declarations,[83] EU legislation [84,85], national legislation [86–98] ethical regulations, data protection and the professional code of conduct of each participating countries. Conditions of study procedure, risk and benefit evaluation, confidence and privacy, and informed consent were approved by the ethical committees in the participating countries. In addition, confidentiality agreements were signed with all participating institutions. Informed consent is obtained from every interviewee, after being informed that participation is voluntary and that they are free to refuse to participate without any negative consequence. Data are coded and processed in such a way that the individual cannot be identified, and appropriately stored. The project and the data processing comply with the European Union Data Protection Legislation and national legislation.

Dissemination

Translating health research findings into practice and policy and disseminating them to the greater public in order to increase the impact on health system performance is an important challenge for any research. To tackle it, a comprehensive and efficient approach is adopted to maximize the research impact during and beyond the project, that will include strategies for

For peer review only

For peer review only - http://bmjopen.bmj.com/site/about/guidelines.xhtml

dissemination and exploitation of results, communication and capacity building, as a transversal component of the project. A key element in it is the participatory approach applied at different levels, including policy, management, healthcare practice and research. Participatory research promotes healthcare practice and policy changes by bringing together the needs of the community and/or health services, scientific evidence, and decision makers, and co-producing knowledge with stakeholders who have the influence and decision-making capacity to implement the knowledge generated through research[99]. EquityCancer-LA expected impact will be achieved, in addition to involving key social actors, through the wide dissemination of results using the mechanisms, methods and materials appropriate to different audiences, as contained in the plan of dissemination: 1) to ensure that results inform policy making to improve the early diagnosis of cancer: a) policy briefs, short reports and guidelines. Moreover, a framework and strategy for the large-scale implementation of the intervention applicable in diverse contexts will be produced, as well as e-tools to improve clinical practice on diagnosis of cancer; b) workshops and webinars to present key findings and policy recommendations to local and national interest groups in the three Latin-American countries; c) building networks of key contacts (academic, governmental, non-governmental, civil society, including users' organisations etc.) in the participating countries and other countries in Latin America and elsewhere, and with international agencies, such as PAHO/WHO, EC, IAEA, IARC, LA Cancer Institute Network, and coordinating with established networks such as the Global Alliance for Chronic Diseases (GACD) network; 2) for dissemination among the academic communities, research papers in open access peer-reviewed national and international journals and other relevant publications, as well as participation in national and international conferences; 3) website, external channels and media to promote visibility and awareness of the project.

FUNDING

For peer review only - http://bmjopen.bmj.com/site/about/guidelines.xhtml

This project has received funding from the European Union's Horizon 2020 research and innovation programme under grant agreement No 965226 on the call topic SC1-BHC-17-2020, Global Alliance for Chronic Diseases - Prevention and/or early diagnosis of cancer.

AUTHORS' CONTRIBUTIONS

M-LV, IV, PE and ASM-P conceived the concept, objectives, and the study design; M-LV and IV were responsible for the development of the study protocol, which received the contributions of the other authors (MR-V, IA-L, A-LT, AP, SD, SSJ). IV and M-LV wrote the first draft of the paper. All authors reviewed the draft versions, made contributions and approved the final version of the article. The authors alone are responsible for the content of this paper.

COMPETING INTEREST

The authors declare that they have no competing interests.

PATIENT CONSENT

Obtained

ETHICS APPROVAL

Comité Ético de Investigación Clínica (CEIC) del Parc de Salut Mar, Barcelona, Spain. Comité de Ética de Investigación con medicamentos (CEIm) de la Fundació Sant Joan de Déu, Barcelona, Spain. Comité de Ética de la Investigación de Salud Metropolitano Norte (CEI-SSMN), Santiago de Chile, Chile. Comité de Ética en Investigación de la Universidad del Rosario (CEI-UR), Bogotá, Colombia. Registration No.: 11878848 in Information, Research and Innovation Systems of the Universidad del Rosario. Comité de Ética de la Investigación en Seres Humanos CEISH-PUCE de la Pontificia Universidad Católica del

For peer review only - http://bmjopen.bmj.com/site/about/guidelines.xhtml

Ecuador (PUCE), Quito, Ecuador. Registration No.: 030-UIO-2021 in Research Register of the PUCE.

REFERENCES

1 De Lemos LLP, De Souza MC, Moreira DP, *et al.* Stage at diagnosis and stage-specific survival of breast cancer in Latin America and the Caribbean: A systematic review and metaanalysis. PLoS One. 2019;**14**. doi:10.1371/journal.pone.0224012

2 Goss PE, Lee BL, Badovinac-Crnjevic T, *et al.* Planning cancer control in Latin America and the Caribbean. *Lancet Oncol* 2013;**14**:391–436. doi:10.1016/S1470-2045(13)70048-2

3 Unger-Saldaña K. Challenges to the early diagnosis and treatment of breast cancer in developing countries. *World J Clin Oncol* 2014;**5**:465–77. doi:10.5306/wjco.v5.i3.465

4 Pinto JA, Pinillos L, Villarreal-Garza C, *et al.* Barriers in Latin America for the management of locally advanced breast cancer. *Ecancermedicalscience* 2019;**13**:897. doi:10.3332/ecancer.2019.897

5 World Health Organization. Guide to cancer early diagnosis. Geneva: 2017. https://apps.who.int/iris/handle/10665/254500

6 World Health Organization. WHO report on cancer: setting priorities, investing wisely and providing care for all. Geneva: 2020. https://www.who.int/publications/i/item/9789240001299

7 IARC. *World cancer report 2014*. 2014. http://publications.iarc.fr/Non-Series-Publications/World-Cancer-Reports/World-Cancer-Report-2014

8 Mazidimoradi A, Hadavandsiri F, Momenimovahed Z, *et al.* Impact of the COVID-19 Pandemic on Colorectal Cancer Diagnosis and Treatment: a Systematic Review. J. Gastrointest. Cancer. 2021. doi:10.1007/s12029-021-00752-5

For peer review only - http://bmjopen.bmj.com/site/about/guidelines.xhtml

9 Dhada S, Stewart D, Cheema E, *et al.* Cancer services during the covid-19 pandemic: Systematic review of patient's and caregiver's experiences. *Cancer Manag Res* 2021;**13**:5875–87. doi:10.2147/CMAR.S318115

10 Jones D, Neal RD, Duffy SRG, *et al.* Impact of the COVID-19 pandemic on the symptomatic diagnosis of cancer: the view from primary care. *Lancet Oncol* 2020;**S1470-2045**:30242–4. doi:10.1016/S1470-2045(20)30242-4

11 Weller D, Vedsted P, Rubin G, *et al.* The Aarhus statement: improving design and reporting of studies on early cancer diagnosis. *Br J Cancer* 2012;**106**:1262–7. doi:10.1038/bjc.2012.68

12 Aday LA, Andersen R. A framework for the study of access to medical care. *Health Serv Res* 1974;**9**:208–20.

13 Piñeros M, Sánchez R, Perry F, *et al.* [Delay for diagnosis and treatment of breast cancer in Bogotá, Colombia]. *Salud Publica Mex* 2011;**53**:478–85.

14 Sardi A, Orozco-Urdaneta M, Velez-Mejia C, *et al.* Overcoming barriers in the implementation of programs for breast and cervical cancers in Cali, Colombia: A pilot model. *J Glob Oncol* 2019;**5**:1–9. doi:10.1200/jgo.19.00054

15 Brand NR, Qu LG, Chao A, *et al.* Delays and barriers to cancer care in low-and middle-income countries: A systematic review. *Oncologist* 2019;**24**:e1371–80. doi:10.1634/theoncologist.2019-0057

16 Vázquez Navarrete ML, Vargas Lorenzo I, Mogollón-Pérez AS, *et al. Redes integradas de servicios de salud en Colombia y Brasil: estudio de casos*. Bogotá, Colombia: Editorial Universidad del Rosario 2017. doi:10.12804/tm9789587388121

17 Murillo R. Social inequalities in cancer in Latin America. In: Vaccarella S, Lortet-Tieulent J, Saracci R, et al. editors, ed. *Reducing Social Inequalities in Cancer: Evidence and Priorities for Research. IARC Scientific Publications, No. 168*. Lyon

For peer review only

For peer review only - http://bmjopen.bmj.com/site/about/guidelines.xhtml

(France): International Agency for Research on Cancer 2019. 223–6.

18 Aguiar PN, Stock GT, Lopes G de L, *et al.* Disparities in cancer epidemiology and care delivery among Brazilian indigenous populations. *Einstein (Sao Paulo)* 2016;**14**:330–7. doi:10.1590/S1679-45082016AO3754

19 Emery JD, Shaw K, Williams B, *et al.* The role of primary care in early detection and follow-up of cancer. *Nat Rev Clin Oncol* 2014;**11**:38–48. doi:10.1038/nrclinonc.2013.212

20 Hamilton W, Walter FM, Rubin G, *et al.* Improving early diagnosis of symptomatic cancer. *Nat Rev Clin Oncol* 2016;**13**:740–9. doi:10.1038/nrclinonc.2016.109

21 Rubin G, Gildea C, Wild S, *et al.* Assessing the impact of an English national initiative for early cancer diagnosis in primary care. *Br J Cancer* 2015;**112 Suppl**:S57-64. doi:10.1038/bjc.2015.43

22 Giovanella L, de Almeida PF, Romero R V, *et al.* [Overview of Primary Health Care in South America: conceptions, components and challenges]. *Saude debate* 2015;**39**:301–22.

23 Rubin G, Berendsen A, Crawford SM, *et al.* The expanding role of primary care in cancer control. *Lancet Oncol* 2015;**16**:1231–72. doi:10.1016/S1470-2045(15)00205-3

24 Albreht T, Martin-Moreno JM, Jelenc M, *et al.* *European Guide for Quality National Cancer Control Programmes*. www.nijz.si

25 Neal RD, Din NU, Hamilton W, *et al.* Comparison of cancer diagnostic intervals before and after implementation of NICE guidelines: Analysis of data from the GBR General Practice Research Database. *Br J Cancer* 2014;**110**:584–92. doi:10.1038/bjc.2013.791

26 Olesen F, Hansen RP, Vedsted P. Delay in diagnosis: The experience in Denmark. *Br J Cancer* 2009;**101**:S5–8. doi:10.1038/sj.bjc.6605383

For peer review only - http://bmjopen.bmj.com/site/about/guidelines.xhtml

27 Prades J, Espinás J, Font R, *et al.* Implementing a Cancer Fast-track Programme between primary and specialised care in Catalonia (Spain): A mixed methods study. *Br J Cancer* 2011;**105**:753–9. doi:10.1038/bjc.2011.308

28 Perez-Bustos AH, Orozco-Urdaneta M, Erazo R, *et al.* A patient navigation initiative to improve access to breast cancer care in Cali, Colombia. *Cancer Rep* 2021;:e1564. doi:https://doi.org/10.1002/cnr2.1564

29 Matassini-Eyzaguirre SM, Figueroa-Montes LE. Navegación de pacientes con sospecha de cáncer de mama: un estudio cualitativo en Lima, Perú. *Rev del Cuerpo Médico Hosp Nac Almanzor Aguinaga Asenjo* 2021;**14**:452–8. doi:10.35434/rcmhnaaa.2021.144.1320

30 Congreso de la República de Colombia. Ley 1384 de 2010. Ley Sandra Ceballos, por la cual se establecen las acciones para la atención integral del cáncer en Colombia. 2010.

31 Republica de Colombia, Ministerio de Protección Social. Plan nacional para el control del cáncer en Colombia 2010-2019. Bogotá, Colombia: 2010. https://www.minsalud.gov.co/sites/rid/Lists/BibliotecaDigital/RIDE/IA/INCA/plan-nacional-control-cancer-2012-2020.pdf

32 Ministerio de Salud. Plan nacional de cáncer Chile 2018-2028. Santiago de Chile, Chile: 2018. https://www.minsal.cl/wp-content/uploads/2019/01/2019.01.23_PLAN-NACIONAL-DE-CANCER_web.pdf

33 Ministerio de Salud. Chile. Ley 21.25 Crea la Ley nacional del Cáncer. 2020.

34 Ministerio de Salud Pública del Ecuador. Estrategia Nacional para la Atención Integral del Cáncer en el Ecuador. 2017;:63.

35 Ministerio de Salud y Protección Social. Colombia. Resolución 3280 de 2018. 2018.

36 del Castillo SM C, Cabrera C ME, Derio P L, *et al.* Resultados del tratamiento del cáncer de mama, programa nacional de cáncer del adulto. *Rev Med Chil*

For peer review only

2017;**145**:1507–13. doi:10.4067/s0034-98872017001201507

37 Murcia E, Aguilera J, Wiesner C, *et al.* Oncology services supply in Colombia. *Colomb Med* 2018;**49**:89–96. doi:10.25100/cm.v49i1.3620

38 Suárez Sánchez F, Quintero Posada A. [Preliminary concepts on oncology services in Colombia]. *Rev Colomb Cancerol* 2008;**12**:12–22.

39 Migowski A, Kneipp Dias MB, Nadanovsky P, *et al.* Guidelines for early detection of breast cancer in Brazil. III – Challenges for implementation. *Cad Saude Publica* 2018;**34**:e00046317. doi:10.1590/0102-311X00046317

40 Torres D, Rashid MU, Gil F, *et al.* Guía de práctica clínica (GPC) para la detección temprana, tratamiento integral, seguimiento y rehabilitación del cáncer de mama. *Breast Cancer Res Treat* 2013;**12 Suppl 1**:S54-69. doi:10.1007/s10549-006-9370-1

41 Migowski A, Azevedo E Silva G, Dias MBK, *et al.* Guidelines for early detection of breast cancer in Brazil. II – New national recommendations, main evidence, and controversies. *Cad Saude Publica* 2018;**34**:1–15. doi:10.1590/0102-311X00074817

42 Duggan C, Dvaladze AL, Tsu V, *et al.* Resource-stratified implementation of a community-based breast cancer management programme in Peru. *Lancet Oncol* 2017;**18**:e607–17. doi:10.1016/S1470-2045(17)30592-2

43 Mansell G, Shapley M, Jordan JL, *et al.* Interventions to reduce primary care delay in cancer referral: A systematic review. *Br J Gen Pract* 2011;**61**:e821-35. doi:10.3399/bjgp11X613160

44 Schichtel M, Rose PW, Sellers C. Educational interventions for primary healthcare professionals to promote the early diagnosis of cancer: a systematic review. *Educ Prim Care* 2013;**24**:274–90. doi:10.1080/14739879.2013.11494186

45 Jensen H, Tørring ML, Olesen F, *et al.* Cancer suspicion in general practice, urgent referral and time to diagnosis: A population-based GP survey and registry study. *BMC*

For peer review only - http://bmjopen.bmj.com/site/about/guidelines.xhtml

Cancer 2014;**14**:636. doi:10.1186/1471-2407-14-636

46 Bradley SH, Kennedy MPT, Neal RD. Recognising Lung Cancer in Primary Care. Adv. Ther. 2019. doi:10.1007/s12325-018-0843-5

47 Spronk I, Korevaar JC, Burgers JS, *et al.* Review of guidance on recurrence risk management for general practitioners in breast cancer, colorectal cancer and melanoma guidelines. Fam. Pract. 2017. doi:10.1093/fampra/cmw140

48 Toftegaard BS, Bro F, Falborg AZ, *et al.* Impact of continuing medical education in cancer diagnosis on GP knowledge, attitude and readiness to investigate - A before-after study. *BMC Fam Pract* Published Online First: 2016. doi:10.1186/s12875-016-0496-x

49 Schichtel M, Rose PW, Sellers C. Educational interventions for primary healthcare professionals to promote the early diagnosis of cancer: A systematic review. *Educ Prim Care* 2013;**24**:274–90. doi:10.1080/14739879.2013.11494186

50 Neal RD, Din NU, Hamilton W, *et al.* Comparison of cancer diagnostic intervals before and after implementation of NICE guidelines: Analysis of data from the GBR General Practice Research Database. *Br J Cancer* 2014;**110**:584–92. doi:10.1038/bjc.2013.791

51 Olver IN, Selva-Nagayam S. Evaluation of a telemedicine link between Darwin and Adelaide to facilitate cancer management. *Telemed J* 2000;**6**:213–8. doi:10.1089/107830200415144

52 Doolittle GC, Spaulding AO. Providing Access to Oncology Care for Rural Patients via Telemedicine. *J Oncol Pract* 2006;**2**:228–30. doi:10.1200/jop.2006.2.5.228

53 Taylor M, Khoo K, Saltman D, *et al.* The use of telemedicine to care for cancer patients at remote sites. *J Clin Oncol* 2007;**25**:6538. doi:10.1200/jco.2007.25.18_suppl.6538

For peer review only - http://bmjopen.bmj.com/site/about/guidelines.xhtml

54 Kunkler IH, Rafferty P, Hill D, *et al.* A pilot study of tele-oncology in Scotland. *J Telemed Telecare* 1998;**4**:113–9. doi:10.1258/1357633981932055

55 Sabesan S, Larkins S, Evans R, *et al.* Telemedicine for rural cancer care in North Queensland: Bringing cancer care home. *Aust J Rural Health* 2012;**20**:259–64. doi:10.1111/j.1440-1584.2012.01299.x

56 Rogers EM. Diffusion of innovations. In: Hoffmann V, ed. *Knowledge and Innovation Management*. Hohenheim: Hohenheim University 2007. 37–50. doi:10.4135/9781412994231.n14

57 Gustafson DH, Sainfort F, Eichler M, *et al.* Developing and testing a model to predict outcomes of organizational change. *Health Serv Res* 2003;**38**:751–76. doi:10.1111/1475-6773.00143

58 Greenhalgh T, Robert G, Macfarlane F, *et al.* Diffusion of innovations in service organizations: Systematic review and recommendations. *Milbank Q* 2004;**82**:581–629. doi:10.1111/j.0887-378X.2004.00325.x

59 Durlak JA, DuPre EP. Implementation matters: a review of research on the influence of implementation on program outcomes and the factors affecting implementation. *Am J Community Psychol* 2008;**41**:327–50. doi:10.1007/s10464-008-9165-0

60 Grol R. Personal paper. Beliefs and evidence in changing clinical practice. *BMJ* 1997;**315**:418–21. doi:10.1136/bmj.315.7105.418

61 Hampshire AJ. What is action research and can it promote change in primary care? *J Eval Clin Pr* 2000;**6**:337–43. doi:10.1046/j.1365-2753.2000.00260.x

62 Campbell M, Fitzpatrick R, Haines A, *et al.* Framework for design and evaluation of complex interventions to improve health. *Br Med J* 2000;**321**:694–6. doi:10.1136/bmj.321.7262.694

63 Soh KL, Davidson PM, Leslie G, *et al.* Action research studies in the intensive care

For peer review only

setting: a systematic review. *Int J Nurs Stud* 2011;**48**:258–68. doi:10.1016/j.ijnurstu.2010.09.014

64 Viswanathan M, Ammerman A, Eng E, *et al.* Community-based participatory research: assessing the evidence. *Evid Rep Technol Assess* 2004:1–8.http://www.ncbi.nlm.nih.gov/pubmed/15460504

65 Waterman H, Tillen D, Dickson R, *et al.* Action research: a systematic review and guidance for assessment. *Health Technol Assess* 2001;**5**:iii.

66 Loewenson R, Laurell AC, Hogstedt C, *et al. Participatory action research in health systems: A methods reader*. Equinet 2014.

67 Vargas I, Eguiguren P, Mogollón-Pérez A-S, *et al.* Understanding the factors influencing the implementation of participatory interventions to improve care coordination. An analytical framework based on an evaluation in Latin America. *Health Policy Plan* 2020;**35**:962–72. doi:10.1093/heapol/czaa066

68 Curran GM, Bauer M, Mittman B, *et al.* Effectiveness-implementation hybrid designs: Combining elements of clinical effectiveness and implementation research to enhance public health impact. *Med Care* 2012;**50**:217–26. doi:10.1097/MLR.0b013e3182408812

69 Damschroder LJ, Aron DC, Keith RE, *et al.* Fostering implementation of health services research findings into practice: A consolidated framework for advancing implementation science. *Implement Sci* 2009;**4**:50. doi:10.1186/1748-5908-4-50

70 Yapa HM, Bärnighausen T. Implementation science in resource-poor countries and communities. *Implement Sci* 2018;**13**:154. doi:10.1186/s13012-018-0847-1

71 Vázquez Navarrete ML, Vargas Lorenzo I, Mogollón-Pérez AS, *et al. Redes integradas de servicios de salud en Colombia y Brasil: Estudio de casos*. Editorial Universidad del Rosario 2018.

For peer review only - http://bmjopen.bmj.com/site/about/guidelines.xhtml

72 DePoy E, Gitlin LN, editors. *Introduction to research: Understanding and applying multiple strategies*. 5th Editio. St. Louis: Mosby 2016. https://www.sciencedirect.com/book/9780323261715/introduction-to-research

73 Palinkas LA, Aarons GA, Horwitz S, *et al.* Mixed method designs in implementation research. *Adm Policy Ment Heal Ment Heal Serv Res* 2011;**38**:44–53. doi:10.1007/s10488-010-0314-z

74 Pettigrew AM. The character and significance of strategy process research. *Strateg Manag J* 1992;**13**:5–16. doi:10.1002/smj.4250130903

75 Chamberlain P, Brown CH, Saldana L. Observational measure of implementation progress in community based settings: The Stages of implementation completion (SIC). *Implement Sci* 2011;**6**. doi:10.1186/1748-5908-6-116

76 Walter F, Webster A, Scott S, *et al.* The Andersen Model of Total Patient Delay: a systematic review of its application in cancer diagnosis. *J Heal Serv Res Policy* 2012;**17**:110–8. doi:10.1258/jhsrp.2011.010113

77 Aday LA, Andersen RM. Equity of access to medical care: A conceptual and empirical overview. *Med Care* 1981;**19**:4–27. doi:10.1097/00005650-198112001-00004

78 Andersen RM. Revisiting the behavioral model and access to medical care: does it matter? *J Health Soc Behav* 1995;**36**:1–10. doi:10.2307/2137284

79 Andersen RM. National health surveys and the behavioral model of health services use. *Med Care* 2008;**46**:647–53. doi:10.1097/MLR.0b013e31817a835d

80 Atun R, Monteiro de Andrade LO, Almeida G, *et al.* Health-system reform and universal health coverage in Latin America. *Lancet* 2015;**385**:1230–47. doi:10.1016/S0140-6736(14)61646-9

81 Gutiérrez Ossa J, Restrepo Avendaño R. O pluralismo estruturado de Londoño e Frenk perante a articulação e modulação do Sistema General de Seguridad Social en Salud

For peer review only - http://bmjopen.bmj.com/site/about/guidelines.xhtml

(SGSS) na Colômbia. *Soc y Econ* 2012;:183–204.

82 Vázquez ML, Vargas I, Unger JP, *et al.* Evaluating the effectiveness of care integration strategies in different healthcare systems in Latin America: The EQUITY-LA II quasi-experimental study protocol. *BMJ Open* 2015;**5**:1–10. doi:10.1136/bmjopen-2014-007037

83 World Medical Association. Declaration of Helsinki: Ethical Principles for Medical Research Involving Human Subjects. 2013. doi:10.1001/jama.2013.281053

84 Regulation (EU) 2016/679 of the European Parliament and of the Council of 27 April 2016 on the protection of natural persons regarding the processing of personal data and on the free movement of such data and repealing Directive 95/46/EC (. https://eur-lex.europa.eu/legal-content/EN/TXT/?uri=CELEX%3A02016R0679-20160504

85 Directive 2001/20/EC of the European Parliament and of the Council. On the approximation of the laws, regulations and administrative provisions of the Member States relating to the implementation of good clinical practice in the conduct of clinical trials. 2001. https://www.legislation.gov.uk/eudr/2001/20/introduction

86 Ley Orgánica 3/2018, de 5 de diciembre, de Protección de Datos Personales y garantía de los derechos digitales. 2018. https://www.boe.es/eli/es/lo/2018/12/05/3

87 Lei n.º 21/2014, de 16 de abril. Lei da investigaçao clínica. 2014. https://www.pgdlisboa.pt/leis/lei_mostra_articulado.php?nid=2089&tabela=leis&so_miolo

88 Ministerio de salud Pública. Ecuador. Reglamento 4889/2014, para la aprobación y seguimiento de los Comités de Ética de Investigación en Seres Humanos (CEISH) y de los Comités de Ética Asistenciales para la Salud (CEAS). 2014. https://www.gob.ec/regulaciones/4889-expedir-reglamento-aprobacion-seguimiento-comites-etica-investigacion-seres-humanos-ceish-comites-etica-asistenciales-salud-

For peer review only

For peer review only - http://bmjopen.bmj.com/site/about/guidelines.xhtml

ceas#:~:text=El acuerdo ministerial tiene por,de la Salud (DIS) del

89 Ministerio de Salud Pública. Ecuador. Acuerdo Ministerial 05216/2014, para el manejo de la información confidencial en el Sistema Nacional de Salud. 2014. https://www.salud.gob.ec/wp-content/uploads/2020/04/Reglamento-Investigación-en-salud-RO-504.pdf

90 Ministerio de Salud Pública. Ecuador. Acuerdo Ministerial 3557/2013, para la creación de la Comisión Nacional de Bioética en Salud, CNBS. 2013. https://www.ucuenca.edu.ec/images/cobias/descargas/acuerdo-3557-14-junio-2013-bioetica-creacion-cnbs.pdf

91 Act. nº 502 of 23 may 2018. Act on supplementary provisions to the regulation on the protection of natural persons with regard to the processing of personal data and on the free movement os such data. 2018. https://www.datatilsynet.dk/media/7753/danish-data-protection-act.pdf

92 Ley estatutaria 1581 de 2012, por la cual se dictan disposiciones generales para la protección de datos personales. 2012. https://www.defensoria.gov.co/public/Normograma 2013_html/Normas/Ley_1581_2012.pdf

93 Colombia M de la PSR de. Resolución número 2378 de 2008, por la cual se adoptan las buenas prácticas clínicas para las instituciones que conducen investigación con medicamentos en seres humanos. 2008. https://www.ins.gov.co/Normatividad/Resoluciones/RESOLUCION 2378 DE 2008.pdf

94 República de Colombia.Ministerio de Salud. Resolución número 8430 de 1993, por la cual se establecen las normas científicas, técnicas y administrativas para la investigación en salud. 1993. https://www.minsalud.gov.co/sites/rid/Lists/BibliotecaDigital/RIDE/DE/DIJ/RESOLU

For peer review only - http://bmjopen.bmj.com/site/about/guidelines.xhtml

CION-8430-DE-1993.PDF

95 Ministerio de salud. Subsecretaria de salud pública. Decreto 114/2011, aprueba reglamento de la ley nº 20.120, sobre la investigación científica en el ser humano, su genoma, y prohíbe la clonación humana. 2011. https://www.bcn.cl/leychile/navegar?idNorma=1032919&idParte=9206067&idVersion=

96 Ministerio de salud subsecretaria de salud pública. Ley 20.584/2012, regula los derechos y deberes que tienen las personas en relación con acciones vinculadas a su atención en salud. 2012. https://www.bcn.cl/leychile/navegar?idNorma=1039348

97 Ministerio de salud subsecretaria de redes asistenciales. Decreto 38/2012, aprueba reglamento sobre derechos y deberes de las personas en relación a las actividades vinculadas con su atención de salud. 2012. https://www.bcn.cl/leychile/navegar?idNorma=1047464

98 Ecuador CN. Ley 67/2006, ley orgánica de salud. 2006. https://www.salud.gob.ec/wp-content/uploads/2017/03/LEY-ORGÁNICA-DE-SALUD4.pdf

99 Boaz A, Hanney S, Borst R, *et al.* How to engage stakeholders in research: Design principles to support improvement. *Heal Res Policy Syst* 2018;**16**:60. doi:10.1186/s12961-018-0337-6

ACKNOWLEDGEMENTS

The authors are most grateful to the Local Steering Committees, managers, doctors and other professionals of the healthcare networks and research fellows that are participating in the study and generously share their effort, time and opinions, thereby making it possible. We highly appreciate the contribution of Laura Esteve and Andrea Miranda to the study protocol.

For peer review only - http://bmjopen.bmj.com/site/about/guidelines.xhtml

We thank the European Union's Horizon 2020 research and innovation programme (SC1-BHC-17-2020) for the funding.

COLLABORATORS: List of collaborators of the study who, together with the authors of the paper, formed part of the EquityCancer-LA project led by M.L. Vazquez (mlvazquez@consorci.org): Spain: Aida Oliver, Verónica Espinel, Zahara Fernández (Consortium for Health Care and Social Services of Catalonia), Josep Maria Borràs (Catalonian Cancer Strategy, Department of Health, Government of Catalonia), Montserrat Gil-Girbau, Antoni Serrano-Blanco, Paula Arroyo-Uriarte (Institut de Recerca Sant Joan de Déu); Chile: María Luisa Garmendia, Ana María Oyarce, Camilo Bass, Isabel Guzmán, Andrea Alvarez, Paola González (Public Health School Dr. Salvador Allende Gossens, University of Chile) Isabel Abarca, Rodney Stock, Berta Cerda, (National Cancer Institute), Guillermo Hartwig, Cristopher Tabilo (North Metropolitan Health Service) Carmen Aravena, Gloria Stephens (South Metropolitan Health Service), Ecuador: Iván Dueñas (Public Health Institute, Pontifical Catholic University of Ecuador).

FIGURE LEGEND

Figure 1. EquityCancer- LA conceptual framework for comprehensive evaluation of the intervention to improve early diagnosis of cancer

CA: cancer; Source: authors adapted from Equity-LA II [67].

Figure 2. EquityCancer-LA participatory, mix-methods approach and research design

Figure 3. The multi-component integrated care intervention to improve early diagnosis of selected frequent cancers. EquityCancer-LA

PC: primary care; SC: secondary care; TC: tertiary care

For peer review only

Figure 4. The process of adapting and implementing the intervention to improve early diagnosis of cancer. EquityCancer-LA

SUPPLEMENTAL MATERIAL

Supplemental Figure 1. Theoretical framework for the analysis of pathways to diagnosis of cancer and influencing factors in LA

HCP: Healthcare provider, Source: Modified from Walter et al. (2012) [76] based on Aday&Andersen[12]

For peer review only

For peer review only - http://bmjopen.bmj.com/site/about/guidelines.xhtml

Figure 1. EquityCancer- LA conceptual framework for comprehensive evaluation of the intervention to improve early diagnosis of cancer

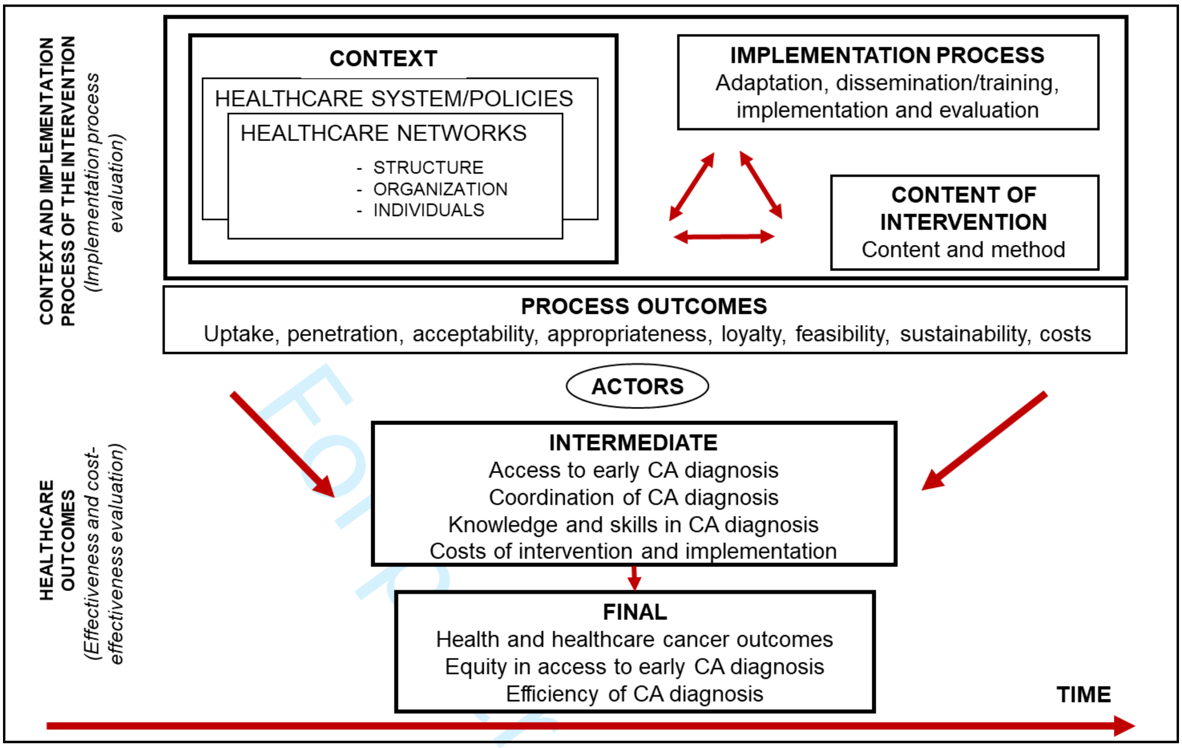

CA: cancer; Source: authors adapted from Equity-LA II [67]

Figure 2. EquityCancer-LA participatory, mix-methods approach and research design

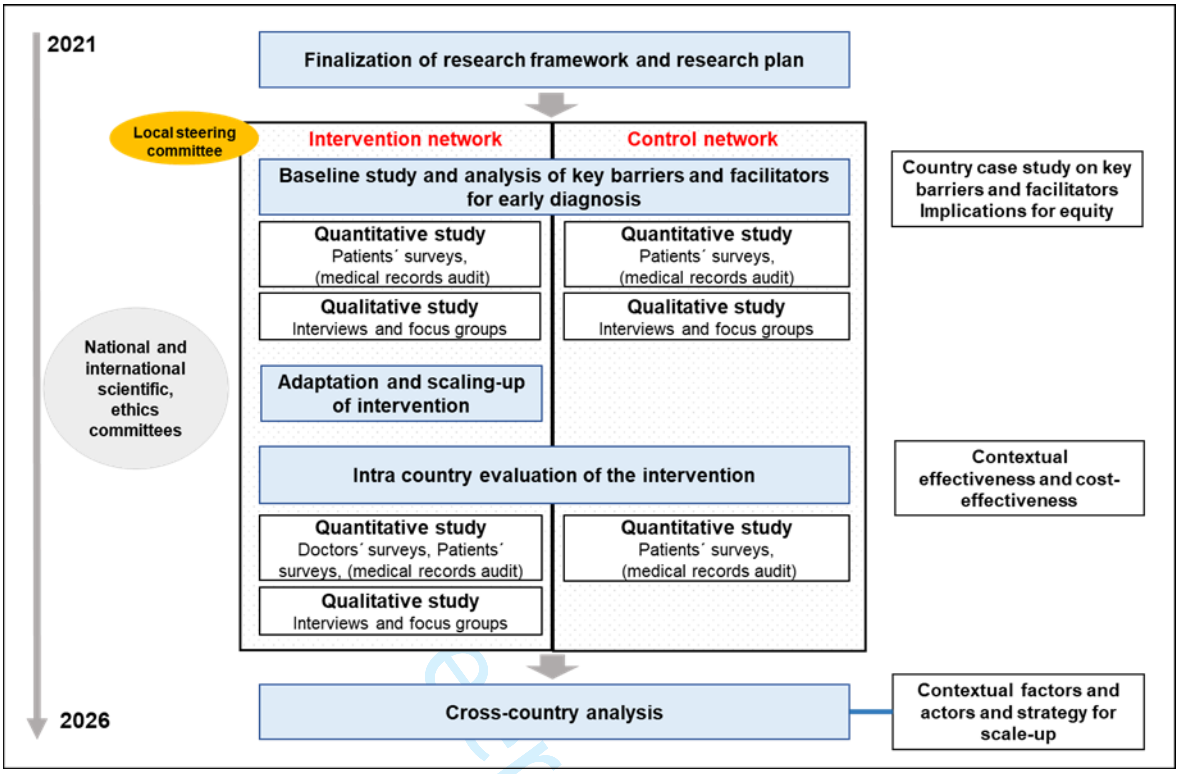

For peer review only

Figure 3. The multi-component integrated care intervention to improve early diagnosis of selected frequent cancers. EquityCancer-LA

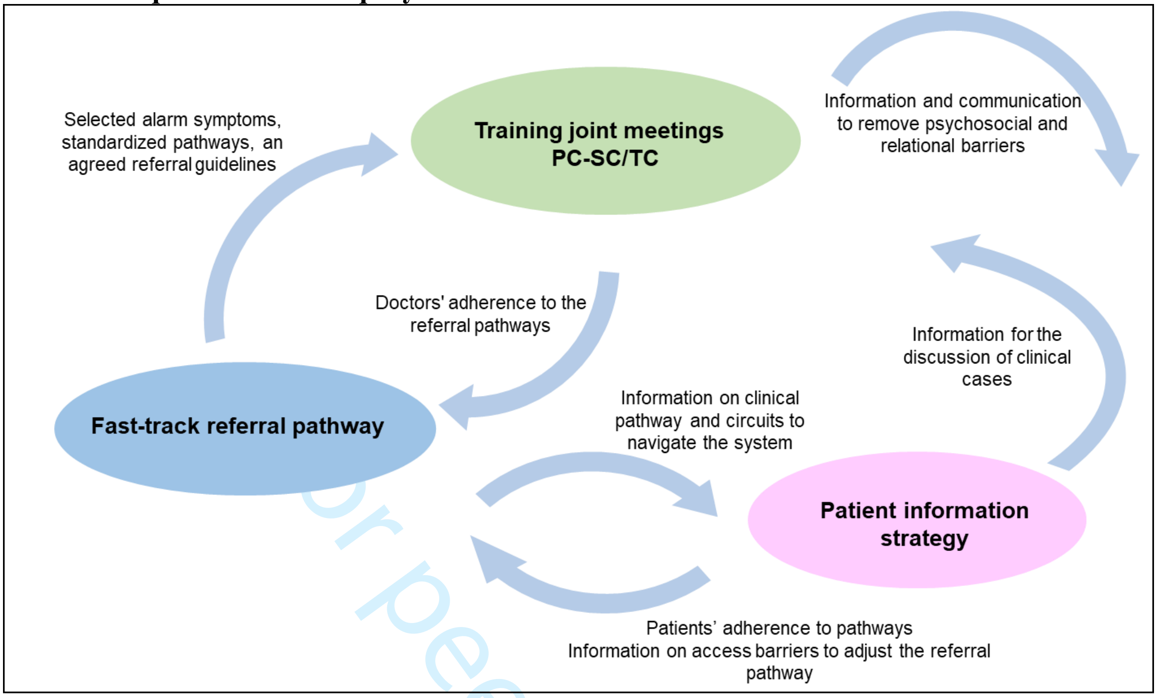

PC: primary care; SC: secondary care; TC: tertiary care

For peer review only

Figure 4. The process of adapting and implementing the intervention to improve early diagnosis of cancer. EquityCancer-LA

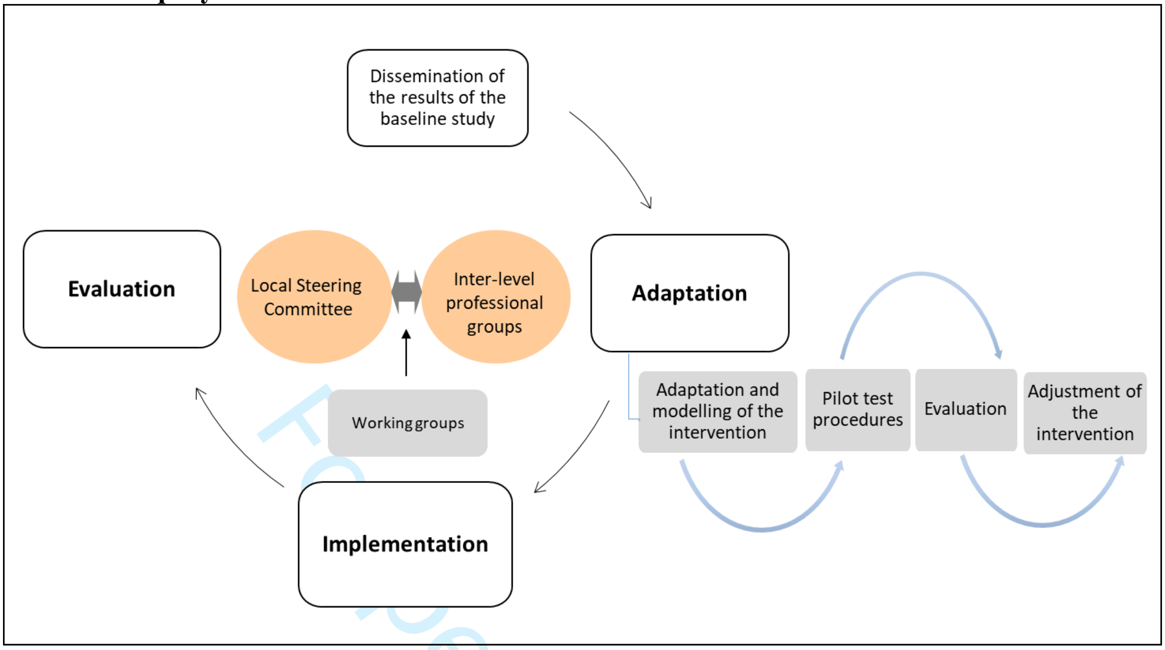

For peer review only

Supplemental Figure 1. Theoretical framework for the analysis of pathways to diagnosis of cancer and influencing factors in LA

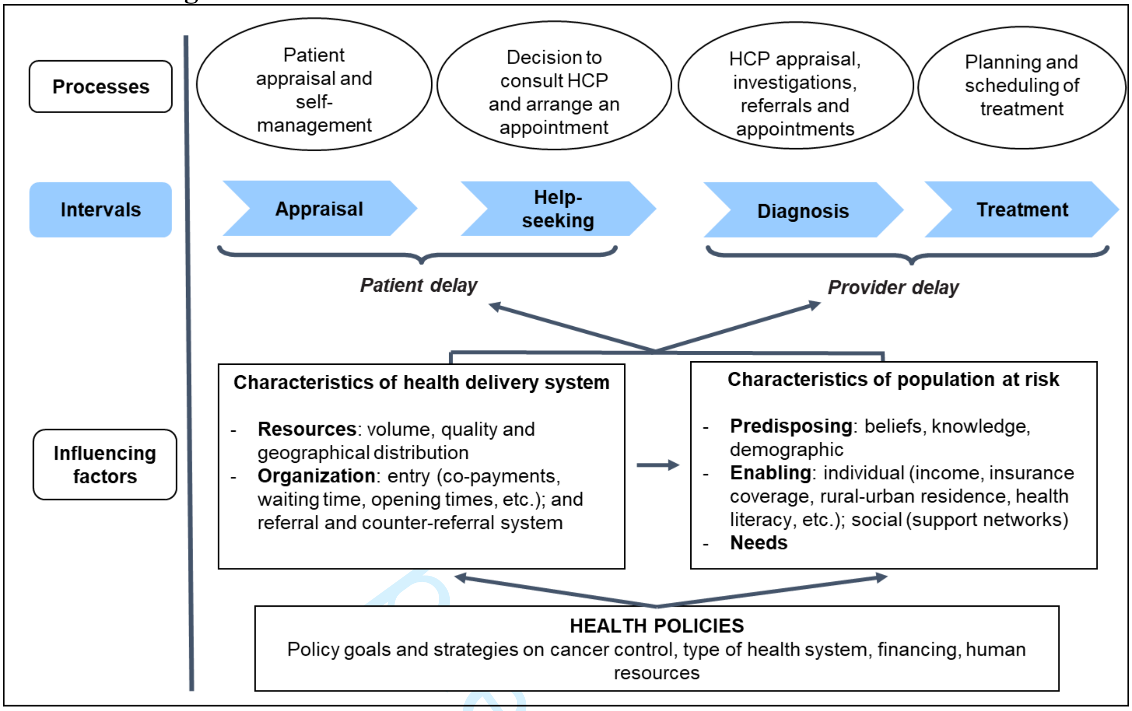

HCP: Healthcare provider, Source: Modified from Walter et al. (2012) [76] based on Aday&Andersen[12]

BMJ Open

Improving equity in access to early diagnosis of cancer in different healthcare systems of Latin America: protocol for the EquityCancer-LA implementation-effectiveness hybrid study

Journal:	*BMJ Open*
Manuscript ID	bmjopen-2022-067439.R1
Article Type:	Protocol
Date Submitted by the Author:	02-Nov-2022
Complete List of Authors:	Vazquez, Maria-Luisa; Research Services and Prospectives on Health Policy of Health and Social Consortium of Catalonia<br>Vargas, Ingrid; Research Services and Prospectives on Health Policy of Health and Social Consortium of Catalonia<br>Rubio-Valera, Maria; Parc Sanitari Sant Joan de Déu, Centro de Investigación Biomédica en Red de Epidemiología y Salud Pública (CIBERESP)<br>Aznar-Lou, Ignacio; Institut de Recerca Sant Joan de Deu, Centro de Investigación Biomédica en Red de Epidemiología y Salud Pública (CIBERESP).Research and Development Unit<br>Eguiguren, Pamela; Universidad de Chile, Escuela de Salud Pública Dr. Salvador Allende. Facultad de Medicina<br>Mogollón-Pérez, Amparo S; Universidad del Rosario, Escuela de Medicina y Ciencias de la Salud<br>Torres-Castillo, Ana Lucía; Pontificia Universidad Católica del Ecuador, Public Health Institute<br>Peralta, Andrés; Pontificia Universidad Católica del Ecuador, Public Health Institute<br>Dias, Sónia; Nova University of Lisbon, Comprehensive Health Research Center (CHRC). NOVA National School of Public Health, Public Health Research Centre<br>Jervelund, Signe; University of Copenhagen, Health Services Research
**Primary Subject Heading**:	Health services research
Secondary Subject Heading:	Oncology, Diagnostics, General practice / Family practice, Public health, Research methods
Keywords:	Change management < HEALTH SERVICES ADMINISTRATION & MANAGEMENT, Organisation of health services < HEALTH SERVICES ADMINISTRATION & MANAGEMENT, Quality in health care < HEALTH SERVICES ADMINISTRATION & MANAGEMENT, MEDICAL EDUCATION & TRAINING, Adult oncology < ONCOLOGY, Health policy < HEALTH SERVICES ADMINISTRATION & MANAGEMENT

Improving equity in access to early diagnosis of cancer in different healthcare systems of Latin America: protocol for the EquityCancer-LA implementation-effectiveness hybrid study

*María-Luisa Vázquez, Ingrid Vargas, Maria Rubio-Valera, Ignacio Aznar-Lou, Pamela Eguiguren, Amparo-Susana Mogollón-Pérez, Ana-Lucía Torres, Andrés Peralta, Sónia Dias, Signe Smith Jervelund, for EquityCancer-LA**

*Collaborators listed at end of article.

Affiliations:

María-Luisa Vázquez
Health Policy and Health Services Research Group, Health Policy Research Unit, Consortium for Health Care and Social Services of Catalonia
Avinguda Tibidabo, 21
08022 Barcelona, Spain
mlvazquez@consorci.org

Ingrid Vargas
Health Policy and Health Services Research Group, Health Policy Research Unit, Consortium for Health Care and Social Services of Catalonia
Avinguda Tibidabo, 21
08022 Barcelona, Spain
ivargas@consorci.org

Maria Rubio-Valera
Centro de Investigación Biomédica en Red de Epidemiología y Salud Pública (CIBERESP)
Parc Sanitari Sant Joan de Déu,
Doctor Antoni Pujadas 42,
Sant Boi de Llobregat, Barcelona, España
maria.rubio@sjd.es

Ignacio Aznar-Lou
Centro de Investigación Biomédica en Red de Epidemiología y Salud Pública (CIBERESP)
Research and Development Unit, Institut de Recerca Sant Joan de Déu
Santa Rosa 39-57
Esplugues de Llobregat, Barcelona, España

For peer review only
For peer review only - http://bmjopen.bmj.com/site/about/guidelines.xhtml

ignacio.aznar@sjd.es

Pamela Eguiguren
Escuela de Salud Pública Dr. Salvador Allende Gossens, Facultad de Medicina, Universidad de Chile
Avenida Independencia, 939
Santiago de Chile, Chile
peguiguren@u.uchile.cl

Amparo-Susana Mogollón-Pérez
Escuela de Medicina y de Ciencias de la Salud, Universidad del Rosario
Carrera 24 No. 63 C-69. Quinta Mutis
Bogotá, Colombia
amparo.mogollon@urosario.edu.co

Ana-Lucía Torres
Public Health Institute, Pontifical Cathloic University of Ecuador
Av. 12 de Octubre, 1076, Vicente Ramón Roca
Quito, Ecuador
atorres331@puce.edu.ec

Andrés Peralta
Public Health Institute, Pontifical Cathloic University of Ecuador
Av. 12 de Octubre, 1076, Vicente Ramón Roca
Quito, Ecuador
aaperalta@puce.edu.ec

Sónia Dias
NOVA National School of Public Health, Public Health Research Centre, NOVA University of Lisbon & Comprehensive Health Research Center (CHRC)
Av. Padre Cruz, 1600-560
Lisboa, Portugal
sonia.dias@ensp.unl.pt

Signe Smith Jervelund
Department of Public Health, Faculty of Health and Medical Sciences, University of Copenhagen
Øster Farimagsgade 5
Copenhagen, Denmark
ssj@sund.ku.dk

Correspondence to:

For peer review only

For peer review only - http://bmjopen.bmj.com/site/about/guidelines.xhtml

Ingrid Vargas
Servei d'Estudis i Prospectives en Polítiques de Salut
Consorci de Salut i Social de Catalunya
Avinguda Tibabo 21
08022 Barcelona
Tel. 00-34-932531820
ivargas@consorci.org

KEYWORDS

Cancer early diagnosis; integrated care interventions; equity in access; implementation research; effectiveness; participatory action research; Latin America

Word count: 4,289

For peer review only

For peer review only - http://bmjopen.bmj.com/site/about/guidelines.xhtml

ABSTRACT

Introduction

Healthcare fragmentation, a main cause for delay in cancer diagnosis and treatment, contributes to high mortality in Latin America (LA), particularly among disadvantaged populations. This research focuses on integrated care interventions, which have been limitedly implemented in the region. The objective is to evaluate the contextual effectiveness of scaling-up an integrated care intervention to improve early diagnosis of frequent cancers in healthcare networks of Chile, Colombia and Ecuador.

Methods and analysis

This research is two-pronged: a) quasi-experimental design (controlled before and after) with an intervention and a control healthcare network in each LA country, using an implementation-effectiveness hybrid approach to assess the intervention process, effectiveness and costs; and b) case study design to analyse access to diagnosis of most frequent cancers. Focussing on the most vulnerable socioeconomic population, it develops in four phases: 1) analysis of delays and barriers to early diagnosis (baseline); 2) intervention adaptation and implementation (primary care (PC) training, fast-track referral pathway and patient information); 3) intra-country evaluation of intervention; and 4) cross-country analysis. Baseline and evaluation studies adopt mixed-methods qualitative (semi-structured individual interviews) and quantitative (patient questionnaire survey) methods. For the latter, a sample size of 174 patients with cancer diagnosis per healthcare network and year was calculated to detect a proportions difference of 15%, before and after intervention (α=0.05; β=0.2) in a two-sided test. A participatory approach will be used to tailor the intervention to each context, led by a local steering committee (professionals, managers, policy-makers, patients and researchers).

Ethics and dissemination

For peer review only - http://bmjopen.bmj.com/site/about/guidelines.xhtml

This study complies with international and national legal stipulations on ethics. It was approved by each country's ethical committee and informed consent will be obtained from participants. Besides the co-production of knowledge with key stakeholders, it will be disseminated through strategies such as policy briefs, workshops, e-tools and scientific papers.

STRENGTHS AND LIMITATIONS OF THIS STUDY

- The study uses implementation-effectiveness hybrid research to generate evidence in Latin America on the effectiveness, implementation process and cost of scaling-up an integrated care intervention to improve early diagnosis of cancer.
- It will utilise comprehensive national and cross-national analysis of time intervals, key barriers and facilitators of access to early cancer diagnosis of the most frequent cancers in LA countries, adopting a common theoretical framework and mixed-method approach.
- The adoption of a participatory approach, involving key stakeholders, will facilitate the adaptation of the intervention to local needs and its uptake, and contribute to overcome contextual barriers and increase sustainability.
- A potential limitation might be the political and managerial changes during the intervention implementation process, leading to limited institutional support for the interventions and its participatory process.
- The absence of a centralised and updated cancer registry in the study areas may hinder and delay the survey data collection.

INTRODUCTION

Delays in diagnosis and treatment contribute to the high and steadily increasing cancer mortality rates in Latin America (LA)[1–4]. WHO recently established early cancer diagnosis

For peer review only - http://bmjopen.bmj.com/site/about/guidelines.xhtml

programmes as a priority for low and middle income countries (LMICs) [5–7] and defined it as the early identification of cancer in patients who have symptoms of the disease [5]. The objective of early cancer diagnosis- which is the focus of EquityCancer-LA- is to identify the disease in people who have symptoms and signs consistent with cancer at the earliest possible opportunity and to link it with treatment without delay [5]. Interventions to ensure adequate and timely access and strong care coordination across the required health services (primary care, diagnostic and specialist services) are key for cancer control. Early diagnosis and referral have been affected by the COVID-19 outbreak responses (confinement, resources diversion and interruption of screening programs, etc.), making interventions in this area more relevant than ever[8–10].

The patient pathway from the discovery of symptoms to a confirmed diagnosis of cancer is often very complicated, encompassing different healthcare levels, services and professionals and contributing to delays in treatment. While delays may differ according to patient and healthcare system characteristics, for its analysis (and to define interventions to address them) two types of intervals are generally distinguished[11]: the *patient interval*, or time taken from the discovery of symptoms to seeking a medical professional, and the *provider interval*, from the request of the first medical consultation to the confirmation of cancer diagnosis.

Delays in cancer diagnosis are accentuated in fragmented healthcare systems such as those in LA, characterized by limited access to health services and poor coordination of care among a wide range of providers. Although further research is required, available studies in LA suggest that most delays occur within the provider interval [4,12,13] and are related to financial, geographic and organizational barriers of access, including poor diagnostic suspicion in primary care (PC) and limited cross-level care coordination [3,14,15]. Population groups most affected are those of low socioeconomic status living in large urban slums, rural

For peer review only - http://bmjopen.bmj.com/site/about/guidelines.xhtml

inhabitants, indigenous populations [3], or those on the subsidized scheme in Colombia (no formal employment)[16], resulting in large inequities in early diagnosis [2,17].

Integrated care as a strategy to improve early cancer diagnosis

The implementation of integrated care interventions focusing on strengthening PC and improving care coordination across care levels are considered essential [18–20]. This is particularly so in PC-based health systems –as in most Latin American countries [21] – in which PC is the entry point to health care and where, around 85% of cancers are diagnosed after symptomatic presentation to PC [18], including cancers for which screening programmes exists [22]. Therefore, to improve the early diagnosis of cancer, PC doctors should be able to assess the probability of cancer, and to refer rapidly suspected cancer patients to specialized care for cancer investigations and treatment [18,22]. To this end, comprehensive multi-component interventions, combining PC training with improved diagnostic and treatment pathways and patient information to navigate the health services, are increasingly recommended [23], also for low and middle income countries (LMICs) [5–7], but introduced mainly in high income countries (HICs) [22,24–28]. In LA, current national cancer plans and strategies [29–34] promote integrated cancer care strategies to address fragmentation and geographic inequity in cancer diagnosis and care. However, the implementation of these integrated care strategies is very limited, their evaluation scarce [35–37] and focused on specific mechanisms [38–41].

The effectiveness of integrated care as a strategy to improve early cancer diagnosis

Available evidence, mostly from HICs, shows that the implementation of continuing education on early diagnosis of PC teams, fast-track referral pathways and guidelines and patient information to navigate the health services are effective [22,25,26,42–44] and efficient [22,45–47] in improving early diagnosis of cancer. Integrated care strategies can improve the appropriateness of referrals and tests and increase diagnostic accuracy for PC doctors

For peer review only - http://bmjopen.bmj.com/site/about/guidelines.xhtml

[22,42,48], which can in turn reduce the provider delay [18,26,49]. Moreover, the use of information and communication technology (ICT) in PC training (e.g. videoconferencing) and virtual clinical consultation show multiple benefits (e.g. increased access to secondary/tertiary care (SC/TC), care coordination across levels, etc.) especially in big cities with traffic problems and rural areas [50–54].

Evidence also shows that interventions are only successful if adapted and implemented taking into account local problems (delays, pathways, barriers) [14], priorities and organizational factors and are agreed upon by the professionals involved across care levels [55–61]. In this respect, participatory and multidisciplinary approaches, including participatory action research (PAR) has developed a growing reputation as an effective means for tailoring interventions and achieving organizational change [62], although its application to improve early diagnosis of cancer has been rare [63]. PAR main characteristics are: non-linear and reflexive process, collaboration between local actors and researchers, involvement of the local stakeholders throughout the process, country research teams as facilitators (capacity building, systematization, monitoring and feedback), and based on three basic principles, egalitarian participation, shared decision-making and co-production of knowledge[64]. Involving main actors lends greater contextual relevance and validity to interventions, creates more interest and increases sustainability [64,65]. Moreover, participatory training methods improve not only knowledge and skills but also favour coordination between professionals and stakeholders throughout the healthcare network[66].

The need for implementation research in Latin America

Implementation research is needed to assess the extent to which an effective intervention for early diagnosis of cancer is also effective in a specific health system and social context. This approach also helps to determine the factors of success or failure in the intervention implementation related to the context and the process, including the keys to its sustainability,

For peer review only - http://bmjopen.bmj.com/site/about/guidelines.xhtml

affordability and large scale use [67,68]. However, limited implementation research has been conducted in LMICs, including LA [66,69] – and even less to evaluate interventions for early diagnosis of cancer. Results of the Equity-LA II implementation research in six LA countries –one of the few carried out in the region so far- highlighted on the one hand, the influence of contextual factors in the implementation of integrated care interventions such as, interference of the political cycle, limited interest and time of professionals. On the other hand, it showed how the participatory approach applied in designing and implementing the interventions enhanced professional motivation to adopt them and continuous institutional support, which ultimately contributed to modifying some contextual barriers [46].

Aim of the EquityCancer-LA project

The aim of EquityCancer-LA is to evaluate the contextual effectiveness of scaling-up a multi-component integrated care intervention to improve early diagnosis of cancer, taking a participatory approach, in healthcare networks of different healthcare systems in Latin America. This project builds upon frameworks, results, and best practices in implementing integrated care interventions using a PAR approach, generated by Equity-LA II[66] research project. This paper outlines the study protocol of EquityCancer-LA.

METHODS AND ANALYSIS

Study design

EquityCancer-LA adopts a double design: a) a quasi-experimental design (a control before and after design) with an intervention and a control health services network in each LA country, using a participatory and implementation-effectiveness hybrid approach[67] to assess the intervention implementation process, contextual effectiveness -i.e. under real-life conditions-, and costs and b) a case study design [70], using mixed-methods [71], for the in-

For peer review only - http://bmjopen.bmj.com/site/about/guidelines.xhtml

depth analysis of key barriers and facilitators to early cancer diagnosis of most frequent cancers and its implications for equity of access.

Analytical frameworks adapted for the evaluation of the intervention effectiveness and analysis of access to early diagnosis of cancer

This study is underpinned by two conceptual frameworks:

I) Analytical framework for the evaluation of an intervention effectiveness and implementation process (Figure 1), based on the Equity-LA II developed framework [66]. It includes a) the evaluation of the intervention effectiveness and cost-effectiveness based on intermediate and final outcomes related to cancer diagnosis; and b) the analysis of the intervention process and how it affects its effectiveness, embracing the three dimensions of Pettigrew and Whipp [68,72]: the *context*, the *process* of design and implementation, and the *content* of the intervention. *Context and process* elements interact in a complex way over time influencing, in the first place, the results of the process of the intervention implementation *(process outcomes*) and ultimately, its intermediate and final outcomes. The most commonly used process outcomes, are those defined by Proctor et al[73]: adoption or uptake, penetration, acceptability, appropriateness, feasibility, intervention costs and sustainability. The effectiveness analysis establishes any significant changes in the outcomes that can be attributed to the integrated care intervention to improve early diagnosis of cancer. Intermediate outcomes refer to patient access to early diagnosis of cancer, to cross-levels care coordination for cancer diagnosis, knowledge of risk factors, associated symptoms and diagnostic ability of participant PC doctors; and the costs of cancer early diagnosis. Final outcomes refer to healthcare and health outcomes (e.g. diagnostic delay; diagnostic stage of cancer), equity and efficiency in early diagnosis and care. The analysis of both contextual factors and the implementation results and costs are carried out taking into account key actors (health professionals, managers and users) viewpoint. The evaluation of the costs of the

For peer review only - http://bmjopen.bmj.com/site/about/guidelines.xhtml

implementation, as part of the analysis of the intervention process, will be oriented by the Stages of implementation completion (SIC) framework[74].II) Framework for the analysis of access to early diagnosis of cancer, built upon the model for the analysis of pathways to cancer diagnosis and treatment by Walter et al. (2012) [75], and incorporating factors influencing healthcare access, as proposed by Aday and Andersen [76–78] (online supplemental Figure 1). According to this framework, delays throughout the patient trajectory to confirmation of cancer diagnosis and initiation of treatment can be influenced by characteristics of the population at risk: need, predisposing (sociodemographic, believes, attitudes, knowledge) and enabling (income, health insurance, residence, etc.) factors, as well as health services' factors: resources availability and organization of health services (fee for service, waiting times, schedules, referral system, etc.) [79]. Health policies can in turn affect access barriers related to the health services or changing (mutable) characteristics of the population at risk [79].

Study context

The field work of EquityCancer-LA is being carried out in three LA countries, Chile, Colombia and Ecuador, and within them, in two public health services networks providing care to middle-low and low income areas. Although the study countries have different health system models, all are segmented by population groups according to socioeconomic or employment status [80,81], with a public subsystem and a private one. The public sector, which is the focus of this study, is financed by social security contributions and/or taxes. It encompasses at least one subsystem dependent on the ministry of health, which is decentralized to different levels of government (departments/provinces and/or municipalities) and is generally aimed at the lower income population and/or those without social security. In each country, two comparable health services networks (one intervention (IN) and one control (CN)) have been selected according to the following criteria: a) provision of a continuum of

For peer review only - http://bmjopen.bmj.com/site/about/guidelines.xhtml

services including at least PC and SC/TC, responsible for diagnosis and treatment of cancer; b) provision of services to a defined population; c) provision of care mainly to urban low and middle low socioeconomic areas; d) willingness to participate and implement the intervention; and e) leadership with competence for implementing the intervention. Selected networks are: in Chile, the northern network (IN) and southern network (CN) of Santiago; in Colombia, central and northern health regions (IN) and southern health region (CN) of Cundinamarca; in Ecuador, 17D06 (IN) and 17D03 (CN) districts in the southern and northern areas of Quito.

Research phases and methods

EquityCancer-LA is structured in four research phases which extends over 60 months (May 2021- April 2026),taking into account Campbell et al. model for the evaluation of complex interventions[82], and keeping with its subsequent update[83], and adopts an interdisciplinary, participatory and mix-methods approach (Figure 2). The phases are: (1) an analysis of key barriers and facilitators for early diagnosis (baseline study), using both qualitative and quantitative methods, which is currently underway; (2) participatory adaptation and implementation of the intervention; (3) monitoring and evaluation of the intervention; and a (4) cross-country comparative analysis. At the beginning of the project, a local steering committee (LSC) of relevant stakeholders involved in the early diagnosis of cancer in the intervention network was established in each country. It is made up of health professionals from different care levels, managers, healthcare insurers (in Colombia), policy-makers nominated by the participating institutions, as well as patients and the research team. The LSC will remain active throughout the project, and will be involved in all research phases, being in charge of the adaptation and implementation of the intervention and dissemination of results.

I. ***Analysis of key barriers and facilitators of early cancer diagnosis*** of the most frequent

For peer review only - http://bmjopen.bmj.com/site/about/guidelines.xhtml

cancers (baseline) ***and implications for equity of access (32 months)***. During the first six months of this phase, the theoretical framework and research plan were finalized, and on the basis of available data, the LSCs selected frequent cancers to be addressed: breast, cervical, colorectal, stomach, lung, prostate, kidney and bladder cancers. The next step is to analyse the access to diagnosis of cancer in the study networks. This analysis employs a mixed methods approach, combining qualitative and quantitative research methods of social sciences for data collection in an iterative and complementary way.

a) A qualitative study to analyse barriers and facilitators of access and patient's pathways to early diagnosis in each particular context from the stakeholders' perspective (19 months). A criterion sample was designed to include all discursive variants on the phenomenon: health professionals, administrative personnel, managers, policy makers, and cancer patients, with particular focus on vulnerable groups (low socioeconomic status, gender, ethnicity, distance to SC/TC services and rurality). Patients are selected from the databases of patients diagnosed with cancer of the health services (in Colombia also from the insurers), and other informants from a list of possible candidates according to the selection criteria, provided by an institutional contact. The final sample size will be reached by saturation of information. Data collection takes place in two phases, before and after the patients' questionnaire survey, using semi-structured individual interviews, and policy documents' analysis. Focus groups with health professionals to explore some issues in depth, might be conducted, if considered necessary and feasible in the pandemic context. Topic guides were developed with one common and one specific section for each informant group. A thematic analysis is being conducted with support of a specialized software (Atlas-ti, NVivo) to identify main contextual barriers and facilitators of cancer diagnosis to inform the adaptation of the intervention in each area. In the second phase, critical cases identified by the patient survey will be invited to become case studies for an

For peer review only - http://bmjopen.bmj.com/site/about/guidelines.xhtml

in-depth analysis to better capture patient journey complexity, reasons for delays in the diagnostic process as well as health consequences.

b) A quantitative study (9 months). A cross-sectional study by means of a questionnaire survey of patients diagnosed of cancer will be conducted in each country to establish differences in use of health services and pathways in cancer diagnosis, time intervals, barriers to early diagnosis among different population groups, costs, and where possible, complemented with PC and SC/TC medical records audits. It will provide also the baseline for the evaluation of the intervention, to compare both networks before and after, and also the intervention and control networks. The study population will comprise patients aged 18 years or more with a confirmed cancer diagnosed and staged in the 3–6 months prior to the survey in the study healthcare networks. Patients with benign tumour, uncertain, with consecutive cancers in same organ (recurrence) or synchronous primary cancers will be excluded. The sample size of the study population has been estimated taking into account the controlled before and after design of the study and in order to ensure the detection of a 15% variation in time intervals, health services use and barriers to the early diagnosis of cancer in a bilateral contrast (before and after, intervention and control). A sample size of 174 patients with a diagnosis of cancer per healthcare network and year has been estimated, on the basis of a power of 80% ($\beta = 0.20$) and a confidence level of 95% ($\alpha = 0.05$). Patients are selected from the databases of patients diagnosed with cancer of the health services (and/or insurers in Colombia). The results will contribute to adapt the intervention to the context, to analyse costs, barriers and inequalities in access to early diagnosis of cancer. The survey started in August 2022.

II. ***Participatory adaptation and implementation of the intervention*** to improve early diagnosis of frequent cancers in the countries of study, under real-life conditions (26 months).

For peer review only - http://bmjopen.bmj.com/site/about/guidelines.xhtml

EquityCancer-LA will adapt, scale-up and assess the feasibility of an effective, innovative and comprehensive integrated care intervention to reduce the provider delay(interval) in the diagnosis of the most frequent cancers. The components of the intervention are: a) *PC doctor's training* to improve PC early diagnosis capacity and care coordination across care levels. According to the context, it will be on-line (videoconferencing) or face-to-face, and reflexive and participatory methods will be used; b) a fast-track referral pathway to expedite referral for diagnostic evaluation of patients jointly designed by PC and SC doctors, supported by the description of alarm symptoms that may raise cancer suspicion, standardised pathways or circuits for the diagnostic process and initial treatment, and referral guidelines agreed upon by PC and SC levels' doctors; and c) a patient information strategy to assist the patients to navigate the system. The three components interact reinforcing each other effectiveness (Figure 3). The intervention has been conceived based on available evidence, WHO recommendations for LMICs [5] and national cancer plans [29–31].

A participatory action research approach (PAR) will be used to tailor the multi-component integrated care intervention to each context and implement it based on the preliminary results of the baseline study and the assessment of the implementation conditions, involving main stakeholders and building on previous Equity-LA II [66,84] experience and methods. A carefully phased approach will be used (Figure 4). The participatory process starts in each LA country with the creation of the LSC. First, the evidence produced on the barriers and facilitators of early diagnosis of cancer and previous experiences related to the intervention in the healthcare networks will discussed with the LSC and the inter-level group of professionals (GP), which will be created with those interested in taking action. The conditions for the intervention implementation (acceptability, appropriateness, feasibility, costs) and contextual barriers and facilitators

For peer review only - http://bmjopen.bmj.com/site/about/guidelines.xhtml

will be evaluated with relevant stakeholders to introduce any necessary changes prior to its implementation. In Chile, an evaluation will be made of the conditions for the extension to the whole network of the joint virtual clinical conferences that were participatory designed and implemented in the framework of the Equity-LA II project.

In the second phase, the different components of the intervention will be adapted by the GP and LSC (contents, method, implementation plan and monitoring indicators) and some of them will be pilot-tested, if relevant. Inter-levels working groups might be established for the adaptation of specific components, made up of participants from the different care levels and departments involved in the cancer care process. In this phase, the definitive plan for the intervention evaluation will be elaborated (including feasible and valid measures of intermediate and final outcomes, etc.).

III. ***Monitoring and evaluation of the intervention*** will address both, the implementation process, as well as, the effectiveness under real-life conditions including the economic evaluation (cost-effectiveness and implementation costs), in each country to determine its sustainability and applicability to other contexts (35 months). *Monitoring the intervention* during the implementation process will facilitate its adaptation, the identification and correction of any contextual factors that may influence its implementation and the costs analysis. Indicators will be elaborated to monitor the implementation process (uptake, costs, etc.) and to identify trends in effectiveness regarding intermediate and, if possible, final outcomes.

The *evaluation of the implementation process* will identify the factors related to the context, process and intervention content that have influenced the implementation and its effectiveness, as well as its sustainability and replicability to other contexts. It will be based mainly on a qualitative study, which will be essential to discriminate what component of the intervention was responsible for what results. Individual interviews and

For peer review only - http://bmjopen.bmj.com/site/about/guidelines.xhtml

focus groups will be conducted with different stakeholders involved in the process. These are: LSC, inter-level group of professionals, managers, policy-makers, and patients. This study will be complemented with quantitative analyses of specific aspects: uptake, acceptability, appropriateness regarding specific problems in cancer early diagnosis (process indicators; doctor survey).

The *evaluation of the effectiveness* will encompass two intra-country comparison (before and after the intervention in each network and between the intervention and control networks). To identify any changes after the intervention, data will be collected by: a) a pre- post- survey to participant doctors, b) the patient survey and medical records audit (if available), using the methods and instruments of the baseline, and c) monitoring indicators. Impact on PC doctor's knowledge will be measured applying an anonymous on-line questionnaire survey before the training starts (baseline), at the end and eight months after to measure any improvement in knowledge. Final outcome measures will be defined after the adaptation and testing of the intervention and may include intermediate healthcare outcomes (pathways in cancer diagnosis, time intervals, barriers of access) and final outcomes (provider delays and stage of cancer at diagnosis).

Two kinds of *economic analysis* will be carried out: a) the implementation cost to estimate total costs of the implementation strategy; and b) a cost-effectiveness analysis of the intervention to calculate incremental costs per reduced diagnostic delay.

The evaluation will be completed combining the results of qualitative and quantitative studies, to identify the effectiveness of the intervention on early diagnosis of cancer and influencing factors in each country.

IV. ***Cross-country comparative analysis*** *(30 months).* Results will be compared across countries to identify contextual factors and actors that influence, on the one hand, access and equity of access to early diagnosis of cancer in different healthcare systems, and on

For peer review only - http://bmjopen.bmj.com/site/about/guidelines.xhtml

the other, the process and effectiveness results of the intervention; and, to inform the strategy to embed the intervention into local, regional and national health policy and practice.

To ensure these components develop according to plan and to an excellent level of quality and maximum societal impact, coordination and management activities, as well as activities for dissemination and exploitation of results and capacity building will run in parallel and will count on the involvement of the LSCs and the support of national and international scientific and international ethics advisory committee.

Patient and public involvement

Patients are part, together with other key stakeholders, of the LSC and WG involved in the adaptation and implementation of the intervention, and specifically, of the component related to the patient information strategy to navigate the health services. They will also be involved in the dissemination of results.

ETHICS AND DISSEMINATION

Ethics approval

Comité Ético de Investigación Clínica (CEIC) del Parc de Salut Mar, Barcelona, Spain. Comité de Ética de Investigación con medicamentos (CEIm) de la Fundació Sant Joan de Déu, Barcelona, Spain. Comité de Ética de la Investigación de Salud Metropolitano Norte (CEI-SSMN), Santiago de Chile, Chile. Comité de Ética en Investigación de la Universidad del Rosario (CEI-UR), Bogotá, Colombia. Registration No.: 11878848 in Information, Research and Innovation Systems of the Universidad del Rosario. Comité de Ética de la Investigación en Seres Humanos CEISH-PUCE de la Pontificia Universidad Católica del

For peer review only - http://bmjopen.bmj.com/site/about/guidelines.xhtml

Ecuador (PUCE), Quito, Ecuador. Registration No.: 030-UIO-2021 in Research Register of the PUCE.

Ethical issues

The development and execution of the project fully complies with all current international conventions and declarations,[85] EU legislation [86,87], national legislation [88,89,98–100,90–97] ethical regulations, data protection and the professional code of conduct of each participating countries. Conditions of study procedure, risk and benefit evaluation, confidence and privacy, and informed consent were approved by the ethical committees in the participating countries. In addition, confidentiality agreements were signed with all participating institutions. Informed consent is obtained from every interviewee, after being informed that participation is voluntary and that they are free to refuse to participate without any negative consequence. Data are coded and processed in such a way that the individual cannot be identified, and appropriately stored. The project and the data processing comply with the European Union Data Protection Legislation and national legislation.

Dissemination

Translating health research findings into practice and policy and disseminating them to the greater public in order to increase the impact on health system performance is an important challenge for any research. To tackle it, a comprehensive and efficient approach is adopted to maximize the research impact during and beyond the project, that will include strategies for dissemination and exploitation of results, communication and capacity building, as a transversal component of the project. A key element in it is the participatory approach applied at different levels, including policy, management, healthcare practice and research. Participatory research promotes healthcare practice and policy changes by bringing together the needs of the community and/or health services, scientific evidence, and decision makers, and co-producing knowledge with stakeholders who have the influence and decision-making

For peer review only

capacity to implement the knowledge generated through research[101]. EquityCancer-LA expected impact will be achieved, in addition to involving key social actors, through the wide dissemination of results using the mechanisms, methods and materials appropriate to different audiences, as contained in the plan of dissemination: 1) to ensure that results inform policy making to improve the early diagnosis of cancer: a) policy briefs, short reports and guidelines. Moreover, a framework and strategy for the large-scale implementation of the intervention applicable in diverse contexts will be produced, as well as e-tools to improve clinical practice on diagnosis of cancer; b) workshops and webinars to present key findings and policy recommendations to local and national interest groups in the three Latin-American countries; c) building networks of key contacts (academic, governmental, non-governmental, civil society, including users' organisations etc.) in the participating countries and other countries in Latin America and elsewhere, and with international agencies, such as PAHO/WHO, EC, IAEA, IARC, LA Cancer Institute Network, and coordinating with established networks such as the Global Alliance for Chronic Diseases (GACD) network; 2) for dissemination among the academic communities, research papers in open access peer-reviewed national and international journals and other relevant publications, as well as participation in national and international conferences; 3) website, external channels and media to promote visibility and awareness of the project.

** ** **

FUNDING

This project has received funding from the European Union's Horizon 2020 research and innovation programme under grant agreement No 965226 on the call topic SC1-BHC-17-2020, Global Alliance for Chronic Diseases - Prevention and/or early diagnosis of cancer.

For peer review only - http://bmjopen.bmj.com/site/about/guidelines.xhtml

CONTRIBUTORS

M-LV, IV, PE and ASM-P conceived the concept, objectives, and the study design; M-LV and IV were responsible for the development of the study protocol, which received the contributions of the other authors (MR-V, IA-L, A-LT, AP, SD, SSJ). IV and M-LV wrote the first draft of the paper. All authors reviewed the draft versions, made contributions and approved the final version of the article. The authors alone are responsible for the content of this paper.

COMPETING INTERESTS

The authors declare that they have no competing interests.

ACKNOWLEDGMENTS

The authors are most grateful to the Local Steering Committees, managers, doctors and other professionals of the healthcare networks and research fellows that are participating in the study and generously share their effort, time and opinions, thereby making it possible. We highly appreciate the contribution of Laura Esteve and Andrea Miranda to the study protocol. We thank the European Union's Horizon 2020 research and innovation programme (SC1-BHC-17-2020) for the funding.

COLLABORATORS

List of collaborators of the study who, together with the authors of the paper, formed part of the EquityCancer-LA project led by M.L. Vazquez (mlvazquez@consorci.org): Spain: Aida Oliver, Verónica Espinel, Zahara Fernández (Consortium for Health Care and Social Services of Catalonia), Josep Maria Borràs (Catalonian Cancer Strategy, Department of Health, Government of Catalonia), Montserrat Gil-Girbau, Antoni Serrano-Blanco, Paula Arroyo-Uriarte (Institut de Recerca Sant Joan de Déu); Chile: María Luisa Garmendia, Ana María Oyarce, Camilo Bass, Isabel Guzmán, Andrea Alvarez, Paola González (Public Health School Dr. Salvador Allende Gossens, University of Chile), Isabel Abarca, Rodney Stock, Berta

For peer review only - http://bmjopen.bmj.com/site/about/guidelines.xhtml

Cerda, (National Cancer Institute), Guillermo Hartwig, Cristopher Tabilo (North Metropolitan Health Service), Carmen Aravena, Gloria Stephens (South Metropolitan Health Service); Colombia: Virginia Garcia, Ana Maria Restrepo, Angela Pinzón, Carol Cardozo, María Camila Rangel, Pablo Cristancho (School of Medicine and Health Sciences, Universidad del Rosario); William Mantilla (La Cardio), Guillermo León, Mauricio O´Byrne, Juan Merchán (Hospital Universitario la Samaritana); Ecuador: Iván Dueñas, Hugo Pereira, Daniel Ruiz, Estefanía Rodríguez (Public Health Institute, Pontifical Catholic University of Ecuador).

REFERENCES

1 De Lemos LLP, De Souza MC, Moreira DP, *et al.* Stage at diagnosis and stage-specific survival of breast cancer in Latin America and the Caribbean: A systematic review and metaanalysis. PLoS One. 2019;**14**. doi:10.1371/journal.pone.0224012

2 Goss PE, Lee BL, Badovinac-Crnjevic T, *et al.* Planning cancer control in Latin America and the Caribbean. *Lancet Oncol* 2013;**14**:391–436. doi:10.1016/S1470-2045(13)70048-2

3 Unger-Saldaña K. Challenges to the early diagnosis and treatment of breast cancer in developing countries. *World J Clin Oncol* 2014;**5**:465–77. doi:10.5306/wjco.v5.i3.465

4 Pinto JA, Pinillos L, Villarreal-Garza C, *et al.* Barriers in Latin America for the management of locally advanced breast cancer. *Ecancermedicalscience* 2019;**13**:897. doi:10.3332/ecancer.2019.897

5 World Health Organization. Guide to cancer early diagnosis. Geneva: 2017. https://apps.who.int/iris/handle/10665/254500

6 World Health Organization. WHO report on cancer: setting priorities, investing wisely and providing care for all. Geneva: 2020.

For peer review only - http://bmjopen.bmj.com/site/about/guidelines.xhtml

https://www.who.int/publications/i/item/9789240001299

7 IARC. *World cancer report 2014*. 2014. http://publications.iarc.fr/Non-Series-Publications/World-Cancer-Reports/World-Cancer-Report-2014

8 Mazidimoradi A, Hadavandsiri F, Momenimovahed Z, *et al.* Impact of the COVID-19 Pandemic on Colorectal Cancer Diagnosis and Treatment: a Systematic Review. J. Gastrointest. Cancer. 2021. doi:10.1007/s12029-021-00752-5

9 Dhada S, Stewart D, Cheema E, *et al.* Cancer services during the covid-19 pandemic: Systematic review of patient's and caregiver's experiences. *Cancer Manag Res* 2021;**13**:5875–87. doi:10.2147/CMAR.S318115

10 Jones D, Neal RD, Duffy SRG, *et al.* Impact of the COVID-19 pandemic on the symptomatic diagnosis of cancer: the view from primary care. *Lancet Oncol* 2020;**S1470-2045**:30242–4. doi:10.1016/S1470-2045(20)30242-4

11 Weller D, Vedsted P, Rubin G, *et al.* The Aarhus statement: improving design and reporting of studies on early cancer diagnosis. *Br J Cancer* 2012;**106**:1262–7. doi:10.1038/bjc.2012.68

12 Piñeros M, Sánchez R, Perry F, *et al.* [Delay for diagnosis and treatment of breast cancer in Bogotá, Colombia]. *Salud Publica Mex* 2011;**53**:478–85.

13 Sardi A, Orozco-Urdaneta M, Velez-Mejia C, *et al.* Overcoming barriers in the implementation of programs for breast and cervical cancers in Cali, Colombia: A pilot model. *J Glob Oncol* 2019;**5**:1–9. doi:10.1200/jgo.19.00054

14 Brand NR, Qu LG, Chao A, *et al.* Delays and barriers to cancer care in low-and middle-income countries: A systematic review. *Oncologist* 2019;**24**:e1371–80. doi:10.1634/theoncologist.2019-0057

15 Vázquez Navarrete ML, Vargas Lorenzo I, Mogollón-Pérez AS, *et al.* *Redes integradas de servicios de salud en Colombia y Brasil: estudio de casos*. Bogotá,

For peer review only - http://bmjopen.bmj.com/site/about/guidelines.xhtml

Colombia: : Editorial Universidad del Rosario 2017. doi:10.12804/tm9789587388121

16 Murillo R. Social inequalities in cancer in Latin America. In: Vaccarella S, Lortet-Tieulent J, Saracci R, et al., editors. *Reducing Social Inequalities in Cancer: Evidence and Priorities for Research. IARC Scientific Publications, No. 168*. Lyon (France): : International Agency for Research on Cancer 2019. 223–6.https://publications.iarc.fr/Book-And-Report-Series/Iarc-Scientific-Publications/Reducing-Social-Inequalities-In-Cancer-Evidence-And-Priorities-For-Research-2019

17 Aguiar PN, Stock GT, Lopes G de L, *et al.* Disparities in cancer epidemiology and care delivery among Brazilian indigenous populations. *Einstein (Sao Paulo)* 2016;**14**:330–7. doi:10.1590/S1679-45082016AO3754

18 Emery JD, Shaw K, Williams B, *et al.* The role of primary care in early detection and follow-up of cancer. *Nat Rev Clin Oncol* 2014;**11**:38–48. doi:10.1038/nrclinonc.2013.212

19 Hamilton W, Walter FM, Rubin G, *et al.* Improving early diagnosis of symptomatic cancer. *Nat Rev Clin Oncol* 2016;**13**:740–9. doi:10.1038/nrclinonc.2016.109

20 Rubin G, Gildea C, Wild S, *et al.* Assessing the impact of an English national initiative for early cancer diagnosis in primary care. *Br J Cancer* 2015;**112 Suppl**:S57-64. doi:10.1038/bjc.2015.43

21 Giovanella L, de Almeida PF, Romero R V, *et al.* [Overview of Primary Health Care in South America: conceptions, components and challenges]. *Saude debate* 2015;**39**:301–22.

22 Rubin G, Berendsen A, Crawford SM, *et al.* The expanding role of primary care in cancer control. *Lancet Oncol* 2015;**16**:1231–72. doi:10.1016/S1470-2045(15)00205-3

23 Albreht T, Martin-Moreno JM, Jelenc M, *et al. European Guide for Quality National*

For peer review only - http://bmjopen.bmj.com/site/about/guidelines.xhtml

Cancer Control Programmes. www.nijz.si

24 Neal RD, Din NU, Hamilton W, *et al.* Comparison of cancer diagnostic intervals before and after implementation of NICE guidelines: Analysis of data from the GBR General Practice Research Database. *Br J Cancer* 2014;**110**:584–92. doi:10.1038/bjc.2013.791

25 Olesen F, Hansen RP, Vedsted P. Delay in diagnosis: The experience in Denmark. *Br J Cancer* 2009;**101**:S5–8. doi:10.1038/sj.bjc.6605383

26 Prades J, Espinás J, Font R, *et al.* Implementing a Cancer Fast-track Programme between primary and specialised care in Catalonia (Spain): A mixed methods study. *Br J Cancer* 2011;**105**:753–9. doi:10.1038/bjc.2011.308

27 Perez-Bustos AH, Orozco-Urdaneta M, Erazo R, *et al.* A patient navigation initiative to improve access to breast cancer care in Cali, Colombia. *Cancer Rep* 2021;:e1564. doi:https://doi.org/10.1002/cnr2.1564

28 Matassini-Eyzaguirre SM, Figueroa-Montes LE. Navegación de pacientes con sospecha de cáncer de mama: un estudio cualitativo en Lima, Perú. *Rev del Cuerpo Médico Hosp Nac Almanzor Aguinaga Asenjo* 2021;**14**:452–8. doi:10.35434/rcmhnaaa.2021.144.1320

29 Congreso de la República de Colombia. Ley 1384 de 2010. Ley Sandra Ceballos, por la cual se establecen las acciones para la atención integral del cáncer en Colombia. 2010.

30 Republica de Colombia, Ministerio de Protección Social. Plan nacional para el control del cáncer en Colombia 2010-2019. Bogotá, Colombia: 2010. https://www.minsalud.gov.co/sites/rid/Lists/BibliotecaDigital/RIDE/IA/INCA/plan-nacional-control-cancer-2012-2020.pdf

31 Ministerio de Salud. Plan nacional de cáncer Chile 2018-2028. Santiago de Chile, Chile: 2018. https://www.minsal.cl/wp-content/uploads/2019/01/2019.01.23_PLAN-

For peer review only - http://bmjopen.bmj.com/site/about/guidelines.xhtml

NACIONAL-DE-CANCER_web.pdf

32 Ministerio de Salud. Chile. Ley 21.25 Crea la Ley nacional del Cáncer. 2020. https://www.bcn.cl/leychile/navegar?idNorma=1149004

33 Ministerio de Salud Pública del Ecuador. Estrategia Nacional para la Atención Integral del Cáncer en el Ecuador. 2017;:63.

34 Ministerio de Salud y Protección Social. Colombia. Resolución 3280 de 2018. 2018. https://www.minsalud.gov.co/Normatividad_Nuevo/Resolución No. 3280 de 20183280.pdf

35 del Castillo SM C, Cabrera C ME, Derio P L, *et al.* Resultados del tratamiento del cáncer de mama, programa nacional de cáncer del adulto. *Rev Med Chil* 2017;**145**:1507–13. doi:10.4067/s0034-98872017001201507

36 Murcia E, Aguilera J, Wiesner C, *et al.* Oncology services supply in Colombia. *Colomb Med* 2018;**49**:89–96. doi:10.25100/cm.v49i1.3620

37 Suárez Sánchez F, Quintero Posada A. [Preliminary concepts on oncology services in Colombia]. *Rev Colomb Cancerol* 2008;**12**:12–22.

38 Migowski A, Kneipp Dias MB, Nadanovsky P, *et al.* Guidelines for early detection of breast cancer in Brazil. III – Challenges for implementation. *Cad Saude Publica* 2018;**34**:e00046317. doi:10.1590/0102-311X00046317

39 Torres D, Rashid MU, Gil F, *et al.* Guía de práctica clínica (GPC) para la detección temprana, tratamiento integral, seguimiento y rehabilitación del cáncer de mama. *Breast Cancer Res Treat* 2013;**12 Suppl 1**:S54-69. doi:10.1007/s10549-006-9370-1

40 Migowski A, Azevedo E Silva G, Dias MBK, *et al.* Guidelines for early detection of breast cancer in Brazil. II – New national recommendations, main evidence, and controversies. *Cad Saude Publica* 2018;**34**:1–15. doi:10.1590/0102-311X00074817

41 Duggan C, Dvaladze AL, Tsu V, *et al.* Resource-stratified implementation of a

For peer review only - http://bmjopen.bmj.com/site/about/guidelines.xhtml

community-based breast cancer management programme in Peru. *Lancet Oncol* 2017;**18**:e607–17. doi:10.1016/S1470-2045(17)30592-2

42 Mansell G, Shapley M, Jordan JL, *et al.* Interventions to reduce primary care delay in cancer referral: A systematic review. *Br J Gen Pract* 2011;**61**:e821-35. doi:10.3399/bjgp11X613160

43 Schichtel M, Rose PW, Sellers C. Educational interventions for primary healthcare professionals to promote the early diagnosis of cancer: a systematic review. *Educ Prim Care* 2013;**24**:274–90. doi:10.1080/14739879.2013.11494186

44 Jensen H, Tørring ML, Olesen F, *et al.* Cancer suspicion in general practice, urgent referral and time to diagnosis: A population-based GP survey and registry study. *BMC Cancer* 2014;**14**:636. doi:10.1186/1471-2407-14-636

45 Bradley SH, Kennedy MPT, Neal RD. Recognising Lung Cancer in Primary Care. Adv. Ther. 2019. doi:10.1007/s12325-018-0843-5

46 Spronk I, Korevaar JC, Burgers JS, *et al.* Review of guidance on recurrence risk management for general practitioners in breast cancer, colorectal cancer and melanoma guidelines. Fam. Pract. 2017. doi:10.1093/fampra/cmw140

47 Toftegaard BS, Bro F, Falborg AZ, *et al.* Impact of continuing medical education in cancer diagnosis on GP knowledge, attitude and readiness to investigate - A before-after study. *BMC Fam Pract* Published Online First: 2016. doi:10.1186/s12875-016-0496-x

48 Schichtel M, Rose PW, Sellers C. Educational interventions for primary healthcare professionals to promote the early diagnosis of cancer: A systematic review. *Educ Prim Care* 2013;**24**:274–90. doi:10.1080/14739879.2013.11494186

49 Neal RD, Din NU, Hamilton W, *et al.* Comparison of cancer diagnostic intervals before and after implementation of NICE guidelines: Analysis of data from the GBR

For peer review only - http://bmjopen.bmj.com/site/about/guidelines.xhtml

General Practice Research Database. *Br J Cancer* 2014;**110**:584–92. doi:10.1038/bjc.2013.791

50 Olver IN, Selva-Nagayam S. Evaluation of a telemedicine link between Darwin and Adelaide to facilitate cancer management. *Telemed J* 2000;**6**:213–8. doi:10.1089/107830200415144

51 Doolittle GC, Spaulding AO. Providing Access to Oncology Care for Rural Patients via Telemedicine. *J Oncol Pract* 2006;**2**:228–30. doi:10.1200/jop.2006.2.5.228

52 Taylor M, Khoo K, Saltman D, *et al.* The use of telemedicine to care for cancer patients at remote sites. *J Clin Oncol* 2007;**25**:6538. doi:10.1200/jco.2007.25.18_suppl.6538

53 Kunkler IH, Rafferty P, Hill D, *et al.* A pilot study of tele-oncology in Scotland. *J Telemed Telecare* 1998;**4**:113–9. doi:10.1258/1357633981932055

54 Sabesan S, Larkins S, Evans R, *et al.* Telemedicine for rural cancer care in North Queensland: Bringing cancer care home. *Aust J Rural Health* 2012;**20**:259–64. doi:10.1111/j.1440-1584.2012.01299.x

55 Rogers EM. Diffusion of innovations. In: Hoffmann V, ed. *Knowledge and Innovation Management*. Hohenheim: : Hohenheim University 2007. 37–50. doi:10.4135/9781412994231.n14

56 Gustafson DH, Sainfort F, Eichler M, *et al.* Developing and testing a model to predict outcomes of organizational change. *Health Serv Res* 2003;**38**:751–76. doi:10.1111/1475-6773.00143

57 Greenhalgh T, Robert G, Macfarlane F, *et al.* Diffusion of innovations in service organizations: Systematic review and recommendations. *Milbank Q* 2004;**82**:581–629. doi:10.1111/j.0887-378X.2004.00325.x

58 Durlak JA, DuPre EP. Implementation matters: a review of research on the influence of

For peer review only

For peer review only - http://bmjopen.bmj.com/site/about/guidelines.xhtml

implementation on program outcomes and the factors affecting implementation. *Am J Community Psychol* 2008;**41**:327–50. doi:10.1007/s10464-008-9165-0

59 Grol R. Personal paper. Beliefs and evidence in changing clinical practice. *BMJ* 1997;**315**:418–21. doi:10.1136/bmj.315.7105.418

60 Hampshire AJ. What is action research and can it promote change in primary care? *J Eval Clin Pr* 2000;**6**:337–43. doi:10.1046/j.1365-2753.2000.00260.x

61 Campbell M, Fitzpatrick R, Haines A, *et al.* Framework for design and evaluation of complex interventions to improve health. *Br Med J* 2000;**321**:694–6. doi:10.1136/bmj.321.7262.694

62 Soh KL, Davidson PM, Leslie G, *et al.* Action research studies in the intensive care setting: a systematic review. *Int J Nurs Stud* 2011;**48**:258–68. doi:10.1016/j.ijnurstu.2010.09.014

63 Viswanathan M, Ammerman A, Eng E, *et al.* Community-based participatory research: assessing the evidence. *Evid Rep Technol Assess* 2004;:1–8.http://www.ncbi.nlm.nih.gov/pubmed/15460504

64 Waterman H, Tillen D, Dickson R, *et al.* Action research: a systematic review and guidance for assessment. *Health Technol Assess* 2001;**5**:iii.

65 Loewenson R, Laurell AC, Hogstedt C, *et al.* *Participatory action research in health systems: A methods reader*. Equinet 2014.

66 Vargas I, Eguiguren P, Mogollón-Pérez A-S, *et al.* Understanding the factors influencing the implementation of participatory interventions to improve care coordination. An analytical framework based on an evaluation in Latin America. *Health Policy Plan* 2020;**35**:962–72. doi:10.1093/heapol/czaa066

67 Curran GM, Bauer M, Mittman B, *et al.* Effectiveness-implementation hybrid designs: Combining elements of clinical effectiveness and implementation research to enhance

For peer review only

For peer review only - http://bmjopen.bmj.com/site/about/guidelines.xhtml

public health impact. *Med Care* 2012;**50**:217–26. doi:10.1097/MLR.0b013e3182408812

68 Damschroder LJ, Aron DC, Keith RE, *et al.* Fostering implementation of health services research findings into practice: A consolidated framework for advancing implementation science. *Implement Sci* 2009;**4**:50. doi:10.1186/1748-5908-4-50

69 Yapa HM, Bärnighausen T. Implementation science in resource-poor countries and communities. *Implement Sci* 2018;**13**:154. doi:10.1186/s13012-018-0847-1

70 DePoy E, Gitlin LN, editors. *Introduction to research: Understanding and applying multiple strategies*. 5th Editio. St. Louis: : Mosby 2016. https://www.sciencedirect.com/book/9780323261715/introduction-to-research

71 Palinkas LA, Aarons GA, Horwitz S, *et al.* Mixed method designs in implementation research. *Adm Policy Ment Heal Ment Heal Serv Res* 2011;**38**:44–53. doi:10.1007/s10488-010-0314-z

72 Pettigrew AM. The character and significance of strategy process research. *Strateg Manag J* 1992;**13**:5–16. doi:10.1002/smj.4250130903

73 Proctor E, Silmere H, Raghavan R, *et al.* Outcomes for implementation research: conceptual distinctions, measurement challenges, and research agenda. *Adm Policy Ment Heal* 2011;**38**:65–76.http://www.ncbi.nlm.nih.gov/pubmed/20957426

74 Chamberlain P, Brown CH, Saldana L. Observational measure of implementation progress in community based settings: The Stages of implementation completion (SIC). *Implement Sci* 2011;**6**. doi:10.1186/1748-5908-6-116

75 Walter F, Webster A, Scott S, *et al.* The Andersen Model of Total Patient Delay: a systematic review of its application in cancer diagnosis. *J Heal Serv Res Policy* 2012;**17**:110–8. doi:10.1258/jhsrp.2011.010113

76 Aday LA, Andersen RM. Equity of access to medical care: A conceptual and empirical

For peer review only

For peer review only - http://bmjopen.bmj.com/site/about/guidelines.xhtml

overview. *Med Care* 1981;**19**:4–27. doi:10.1097/00005650-198112001-00004

77 Andersen RM. Revisiting the behavioral model and access to medical care: does it matter? *J Health Soc Behav* 1995;**36**:1–10. doi:10.2307/2137284

78 Andersen RM. National health surveys and the behavioral model of health services use. *Med Care* 2008;**46**:647–53. doi:10.1097/MLR.0b013e31817a835d

79 Aday LA, Andersen R. A framework for the study of access to medical care. *Health Serv Res* 1974;**9**:208–20.

80 Atun R, Monteiro de Andrade LO, Almeida G, *et al.* Health-system reform and universal health coverage in Latin America. *Lancet* 2015;**385**:1230–47. doi:10.1016/S0140-6736(14)61646-9

81 Gutiérrez Ossa J, Restrepo Avendaño R. O pluralismo estruturado de Londoño e Frenk perante a articulação e modulação do Sistema General de Seguridad Social en Salud (SGSS) na Colômbia. *Soc y Econ* 2012;:183–204.

82 Campbell NC, Murray E, Darbyshire J, *et al.* Designing and evaluating complex interventions to improve health care. *Br Med J* 2007;**334**:455–9. doi:10.1136/bmj.39108.379965.be

83 Skivington K, Matthews L, Simpson SA, *et al.* A new framework for developing and evaluating complex interventions: update of Medical Research Council guidance. doi:10.1136/bmj.n2061

84 Vázquez ML, Vargas I, Unger JP, *et al.* Evaluating the effectiveness of care integration strategies in different healthcare systems in Latin America: The EQUITY-LA II quasi-experimental study protocol. *BMJ Open* 2015;**5**:1–10. doi:10.1136/bmjopen-2014-007037

85 World Medical Association. Declaration of Helsinki: Ethical Principles for Medical Research Involving Human Subjects. 2013. doi:10.1001/jama.2013.281053

For peer review only

86 Regulation (EU) 2016/679 of the European Parliament and of the Council of 27 April 2016 on the protection of natural persons regarding the processing of personal data and on the free movement of such data and repealing Directive 95/46/EC (. https://eur-lex.europa.eu/legal-content/EN/TXT/?uri=CELEX%3A02016R0679-20160504

87 Directive 2001/20/EC of the European Parliament and of the Council. On the approximation of the laws, regulations and administrative provisions of the Member States relating to the implementation of good clinical practice in the conduct of clinical trials. 2001. https://www.legislation.gov.uk/eudr/2001/20/introduction

88 Ley Orgánica 3/2018, de 5 de diciembre, de Protección de Datos Personales y garantía de los derechos digitales. 2018. https://www.boe.es/eli/es/lo/2018/12/05/3

89 Lei n.º 21/2014, de 16 de abril. Lei da investigaçao clínica. 2014. https://www.pgdlisboa.pt/leis/lei_mostra_articulado.php?nid=2089&tabela=leis&so_miolo

90 Act. nº 502 of 23 may 2018. Act on supplementary provisions to the regulation on the protection of natural persons with regard to the processing of personal data and on the free movement os such data. 2018. https://www.datatilsynet.dk/media/7753/danish-data-protection-act.pdf

91 Ley estatutaria 1581 de 2012, por la cual se dictan disposiciones generales para la protección de datos personales. 2012. https://www.defensoria.gov.co/public/Normograma 2013_html/Normas/Ley_1581_2012.pdf

92 Colombia M de la PSR de. Resolución número 2378 de 2008, por la cual se adoptan las buenas prácticas clínicas para las instituciones que conducen investigación con medicamentos en seres humanos. 2008. https://www.ins.gov.co/Normatividad/Resoluciones/RESOLUCION 2378 DE 2008.pdf

For peer review only - http://bmjopen.bmj.com/site/about/guidelines.xhtml

93 República de Colombia.Ministerio de Salud. Resolución número 8430 de 1993, por la cual se establecen las normas científicas, técnicas y administrativas para la investigación en salud. 1993. https://www.minsalud.gov.co/sites/rid/Lists/BibliotecaDigital/RIDE/DE/DIJ/RESOLUCION-8430-DE-1993.PDF

94 Ministerio de salud. Subsecretaria de salud pública. Decreto 114/2011, aprueba reglamento de la ley nº 20.120, sobre la investigación científica en el ser humano, su genoma, y prohíbe la clonación humana. 2011. https://www.bcn.cl/leychile/navegar?idNorma=1032919&idParte=9206067&idVersion=

95 Ministerio de salud subsecretaria de salud pública. Ley 20.584/2012, regula los derechos y deberes que tienen las personas en relación con acciones vinculadas a su atención en salud. 2012. https://www.bcn.cl/leychile/navegar?idNorma=1039348

96 Ministerio de salud subsecretaria de redes asistenciales. Decreto 38/2012, aprueba reglamento sobre derechos y deberes de las personas en relación a las actividades vinculadas con su atención de salud. 2012. https://www.bcn.cl/leychile/navegar?idNorma=1047464

97 Ecuador CN. Ley 67/2006, ley orgánica de salud. 2006. https://www.salud.gob.ec/wp-content/uploads/2017/03/LEY-ORGÁNICA-DE-SALUD4.pdf

98 Ministerio de salud Pública. Ecuador. Reglamento 4889/2014, para la aprobación y seguimiento de los Comités de Ética de Investigación en Seres Humanos (CEISH) y de los Comités de Ética Asistenciales para la Salud (CEAS). 2014. https://www.gob.ec/regulaciones/4889-expedir-reglamento-aprobacion-seguimiento-comites-etica-investigacion-seres-humanos-ceish-comites-etica-asistenciales-salud-ceas#:~:text=El acuerdo ministerial tiene por,de la Salud (DIS) del

For peer review only - http://bmjopen.bmj.com/site/about/guidelines.xhtml

99 Ministerio de Salud Pública. Ecuador. Acuerdo Ministerial 05216/2014, para el manejo de la información confidencial en el Sistema Nacional de Salud. 2014. https://www.salud.gob.ec/wp-content/uploads/2020/04/Reglamento-Investigación-en-salud-RO-504.pdf

100 Ministerio de Salud Pública. Ecuador. Acuerdo Ministerial 3557/2013, para la creación de la Comisión Nacional de Bioética en Salud, CNBS. 2013. https://www.ucuenca.edu.ec/images/cobias/descargas/acuerdo-3557-14-junio-2013-bioetica-creacion-cnbs.pdf

101 Boaz A, Hanney S, Borst R, *et al.* How to engage stakeholders in research: Design principles to support improvement. *Heal Res Policy Syst* 2018;**16**:60. doi:10.1186/s12961-018-0337-6

For peer review only

FIGURE LEGEND

Figure 1. EquityCancer- LA conceptual framework for comprehensive evaluation of the intervention to improve early diagnosis of cancer

Adapted from Equity-LA II [66]. CA: cancer.

Figure 2. EquityCancer-LA participatory, mix-methods approach and research design

Figure 3. The multi-component integrated care intervention to improve early diagnosis of selected frequent cancers. EquityCancer-LA

PC: primary care; SC: secondary care; TC: tertiary care.

Figure 4. The process of adapting and implementing the intervention to improve early diagnosis of cancer. EquityCancer-LA

For peer review only - http://bmjopen.bmj.com/site/about/guidelines.xhtml

SUPPLEMENTAL MATERIAL

Supplemental Figure 1. Theoretical framework for the analysis of pathways to diagnosis of cancer and influencing factors in LA

Adapted from Walter et al. (2012) [75] based on Aday & Andersen[79]. HCP: Healthcare provider.

For peer review only

For peer review only - http://bmjopen.bmj.com/site/about/guidelines.xhtml

Figure 1. EquityCancer- LA conceptual framework for comprehensive evaluation of the intervention to improve early diagnosis of cancer

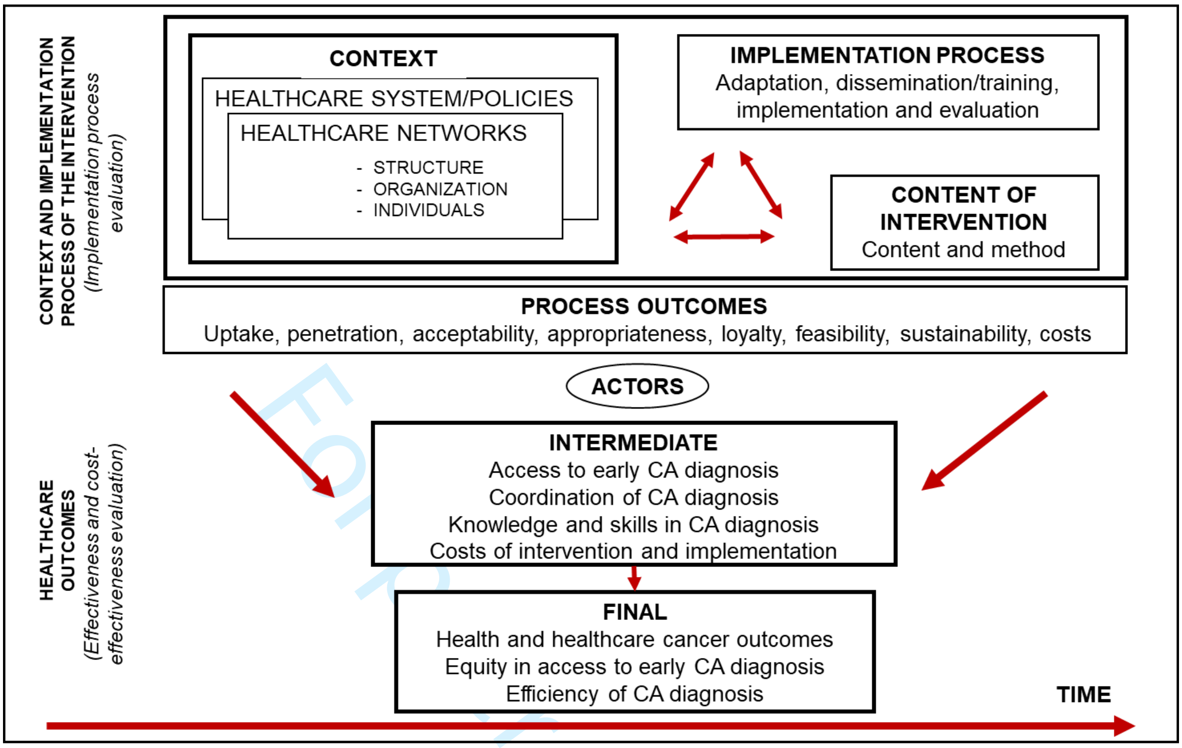

CA: cancer; Source: authors adapted from Equity-LA II [66]

For peer review only

Figure 2. EquityCancer-LA participatory, mix-methods approach and research design

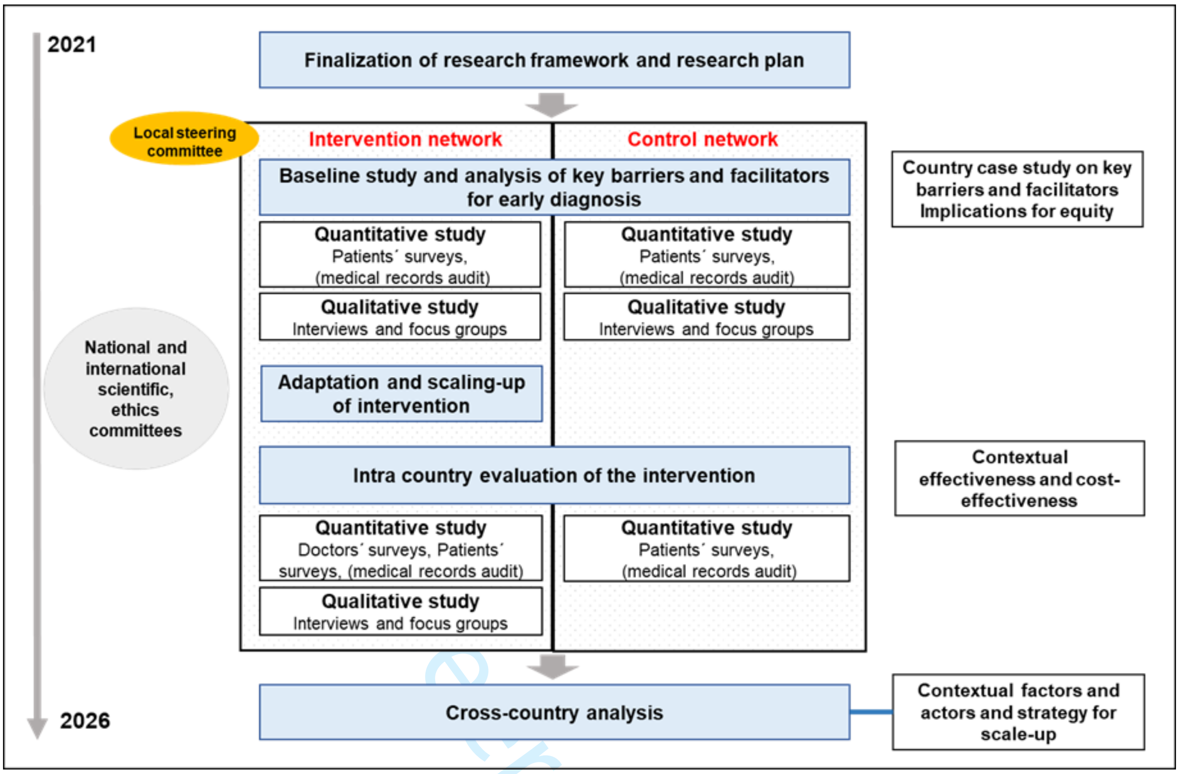

For peer review only

Figure 3. The multi-component integrated care intervention to improve early diagnosis of selected frequent cancers. EquityCancer-LA

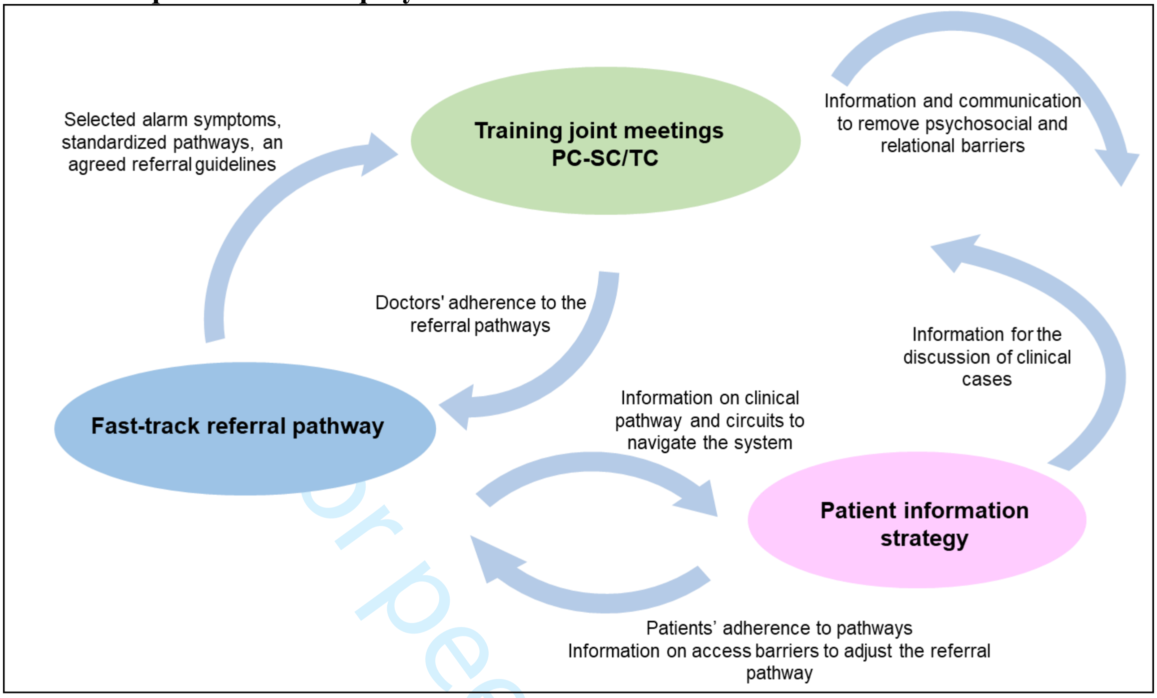

PC: primary care; SC: secondary care; TC: tertiary care

For peer review only

Figure 4. The process of adapting and implementing the intervention to improve early diagnosis of cancer. EquityCancer-LA

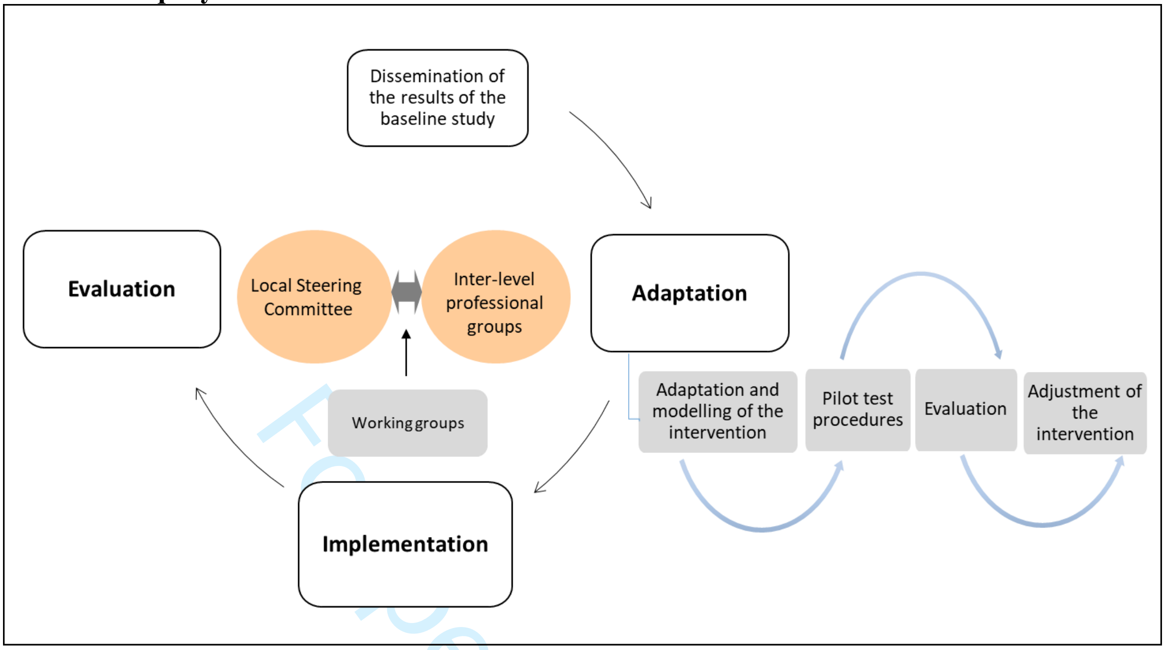

For peer review only

Supplemental Figure 1. Theoretical framework for the analysis of pathways to diagnosis of cancer and influencing factors in LA

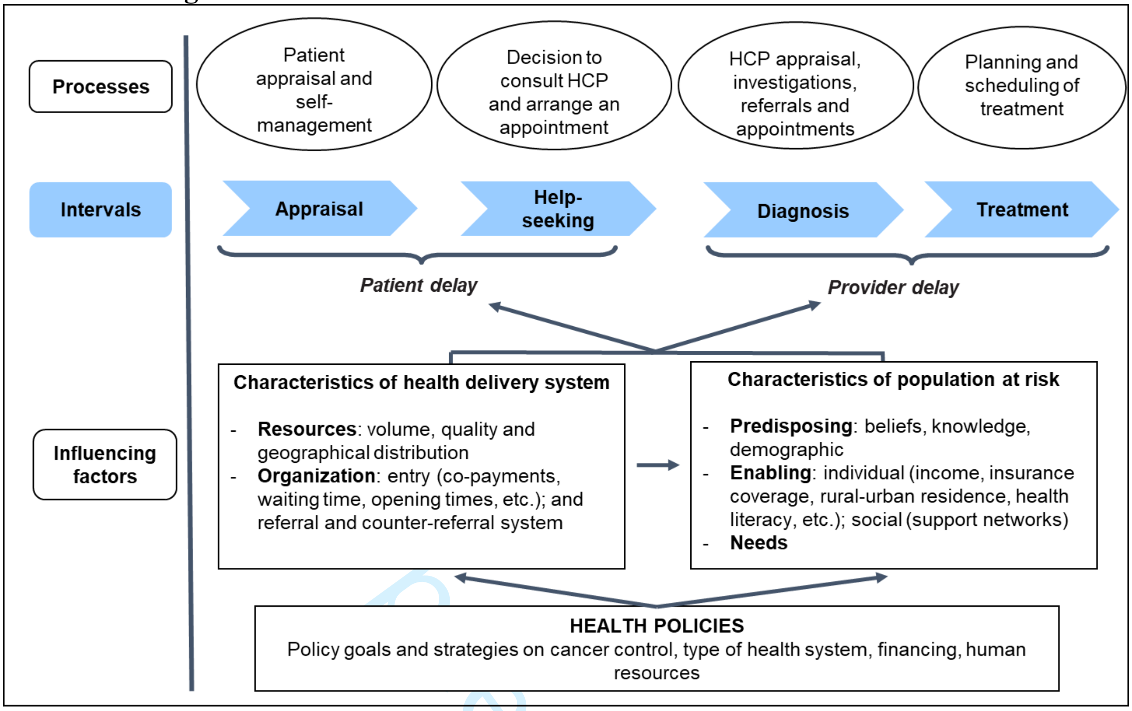

HCP: Healthcare provider, Source: Modified from Walter et al. (2012) [75] based on Aday&Andersen[79]

For peer review only
